# BCG‐Derived Outer Membrane Vesicles Induce TLR2‐Dependent Trained Immunity to Protect Against Polymicrobial Sepsis

**DOI:** 10.1002/advs.202504101

**Published:** 2025-06-24

**Authors:** Yuan Gong, Wenyan Hao, Lingqi Xu, Yingyi Yang, Zhenzhen Dong, Peng Pan, Zhenjiang Bai, Jie Huang, Kaidi Yang, Zhu Jin, Rui Kang, Qiang Shan, Jiang huai Wang, Zijian Zhou, Daolin Tang, Jian Wang, Huiting Zhou

**Affiliations:** ^1^ Institute of Pediatric Research Children's Hospital of Soochow University Suzhou 215025 China; ^2^ Department of Pediatric Surgery Affiliated Hospital of Zunyi Medical University Zunyi Guizhou 563000 China; ^3^ Department of Surgery UT Southwestern Medical Center Dallas TX 75235 USA; ^4^ National Key Laboratory of Immunity and Inflammation Suzhou Institute of Systems Medicine Chinese Academy of Medical Sciences & Peking Union Medical College Suzhou 215123 China; ^5^ Department of Academic Surgery University College Cork Cork University Hospital Cork T12 DC4A Ireland; ^6^ State Key Laboratory of Vaccines for Infectious Diseases Xiang An Biomedicine Laboratory & Center for Molecular Imaging and Translational Medicine National Innovation Platform for Industry‐Education Integration in Vaccine Research School of Public Health Xiamen University Xiamen 361102 China; ^7^ Department of Surgery Children's Hospital of Soochow University Suzhou 215025 China

**Keywords:** BCG, OMVs, sepsis, trained immunity

## Abstract

Trained immunity, an innate immune memory, has emerged as a promising strategy to enhance host defense against sepsis, a leading cause of mortality in critical care. While Bacillus Calmette–Guérin (BCG) is the most widely used vaccine for tuberculosis prevention, its broader use as an inducer of trained immunity is limited by adverse reactions. Here, it is reported that BCG‐derived outer membrane vesicles (B‐OMVs) effectively trigger trained immunity to protect against experimental polymicrobial sepsis. Comprehensive characterization and safety assessments confirmed that B‐OMVs exhibited no significant toxicity or pathological effects, positioning them as a promising alternative to conventional BCG vaccines and *E. coli*‐derived outer membrane vesicles (E‐OMVs) in terms of both efficacy and safety. Mechanistically, B‐OMVs enhanced trained immunity by promoting hematopoietic stem cell expansion and myelopoiesis via toll like receptor 2 (TLR2)‐dependent activation of aerobic glycolysis and epigenetic reprogramming. This led to an amplified immune response and enhanced phagocytic activity in bone marrow‐derived macrophages. Together, these findings establish B‐OMVs as a novel immunomodulatory agent against sepsis‐induced immune dysfunction, with translational potential.

## Graphical abstract

B‐OMVs, nanoscale vesicles derived from BCG, are identified as effective and safe inducers of trained immunity that protect against sepsis. Through TLR2‐mediated metabolic and epigenetic reprogramming of hematopoietic stem cells, B‐OMVs enhance inflammatory response and phagocytic activity. These findings establish B‐OMVs as a novel immunomodulatory agent against sepsis‐induced immune dysfunction, with translational potential.

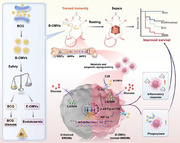

## 1 Introduction

Sepsis remains one of the most significant global health challenges, with ≈49 million cases and over 11 million sepsis‐related deaths annually, accounting for 19.7% of total global mortality.^[^ ^1^, ^2^, ^3^, ^4^ ^]^ Defined as a life‐threatening multi‐organ dysfunction caused by a dysregulated host response to infection, sepsis involves a substantial imbalance between innate and adaptive immune responses.^[^ ^5^ ^]^ This imbalance leads to overwhelming systemic inflammation, disruption of homeostasis, and increased mortality.^[^ ^6^, ^7^, ^8^, ^9^ ^]^ Despite advancements in infection control, hemodynamic resuscitation, and organ support, sepsis‐related mortality remains unacceptably high. This is further exacerbated by the emergence of multidrug‐resistant organisms and the overuse of antibiotics, which complicate treatment strategies and represent a growing public health concern.^[^ ^10^ ^]^ A key contributor to sepsis mortality is the dysregulated host immune response. However, current clinical interventions primarily target pathogen elimination and circulatory support, with limited focus on restoring immune homeostasis. This critical gap remains unaddressed in standard care and is the subject of ongoing investigation.^[^ ^11^, ^12^ ^]^ These considerations highlight the therapeutic potential of immunomodulatory approaches in improving sepsis outcomes.

In recent years, the concept of trained immunity has emerged as an innovative strategy for immune modulation.^[^ ^13^ ^]^ Trained immunity, or innate immune memory, involves extensive metabolic and epigenetic reprogramming that imparts memory characteristics to innate immune cells, such as monocytes, macrophages, natural killer (NK) cells, and innate lymphoid cells (ILCs), following transient stimulation.^[^ ^13^, ^14^ ^]^ Trained immunity enhances protection against secondary infections, aiding in the restoration of immune homeostasis.^[^ ^14^, ^15^ ^]^ Given its potential to strengthen immune defenses against both specific and non‐specific challenges, trained immunity offers a promising therapeutic strategy for sepsis prevention and treatment.^[^ ^16^, ^17^, ^18^ ^]^

Bacillus Calmette–Guérin (BCG), a live attenuated vaccine widely used for tuberculosis prevention, is one of the most established agents for inducing trained immunity.^[^ ^19^, ^20^, ^21^, ^22^, ^23^, ^24^ ^]^ In addition to preventing tuberculosis, BCG has been shown to offer non‐specific protection against various diseases, including cancer, viral infections, and autoimmune diseases.^[^ ^22^, ^24^, ^25^ ^]^ BCG vaccination provides rapid protection against neonatal sepsis by inducing emergency granulopoiesis.^[^ ^26^ ^]^ However, its clinical application is limited due to reactogenicity, including adverse effects such as BCG disease (BCGitis) or disseminated BCG disease (BCGosis), particularly in immunocompromised individuals.^[^ ^27^ ^]^ These safety concerns emphasize the need for safer alternatives that retain the benefits of trained immunity whereas minimizing risks.

Outer membrane vesicles (OMVs) are nanovesicles secreted by bacteria, composed of proteins and lipids.^[^ ^28^ ^]^ Lipoproteins on OMVs act as pathogen‐associated molecular patterns (PAMPs) that activate innate immune responses through pattern recognition receptors (PRRs), particularly toll‐like receptors (TLRs).^[^ ^29^, ^30^ ^]^ These lipoproteins can induce trained immunity,^[^ ^31^, ^32^ ^]^ and OMVs have been considered as vaccine adjuvants and vectors.^[^ ^33^, ^34^, ^35^, ^36^, ^37^ ^]^ However, OMVs often contain endotoxins, such as lipopolysaccharides (LPS), which can trigger both immune responses and reactogenicity, leading to adverse outcomes like intestinal barrier dysfunction.^[^ ^38^ ^]^ Therefore, detoxifying OMVs is essential for their safe therapeutic application. While various detoxification methods have been developed, achieving an optimal balance between minimizing toxicity and maximizing efficacy remains a significant challenge.^[^ ^39^, ^40^ ^]^

To address these limitations, this study developed Bacillus Calmette–Guérin‐derived outer membrane vesicles (B‐OMVs), combining the benefits of both BCG and OMVs. As derivatives of a Gram‐positive bacterium, B‐OMVs are devoid of LPS and non‐replicative, thereby mitigating risks associated with LPS tolerance and infection. Their nanoscale size enhances biodistribution and tissue penetration. We show that B‐OMVs induce trained immunity more effectively than BCG, promoting immune reprogramming and conferring robust protection against sepsis. These findings position B‐OMVs as promising immunotherapeutic candidates for sepsis vaccines and the treatment of infectious diseases.

## 2 Results

### 2.1 Characterization of B‐OMVs

**Figure 1:**
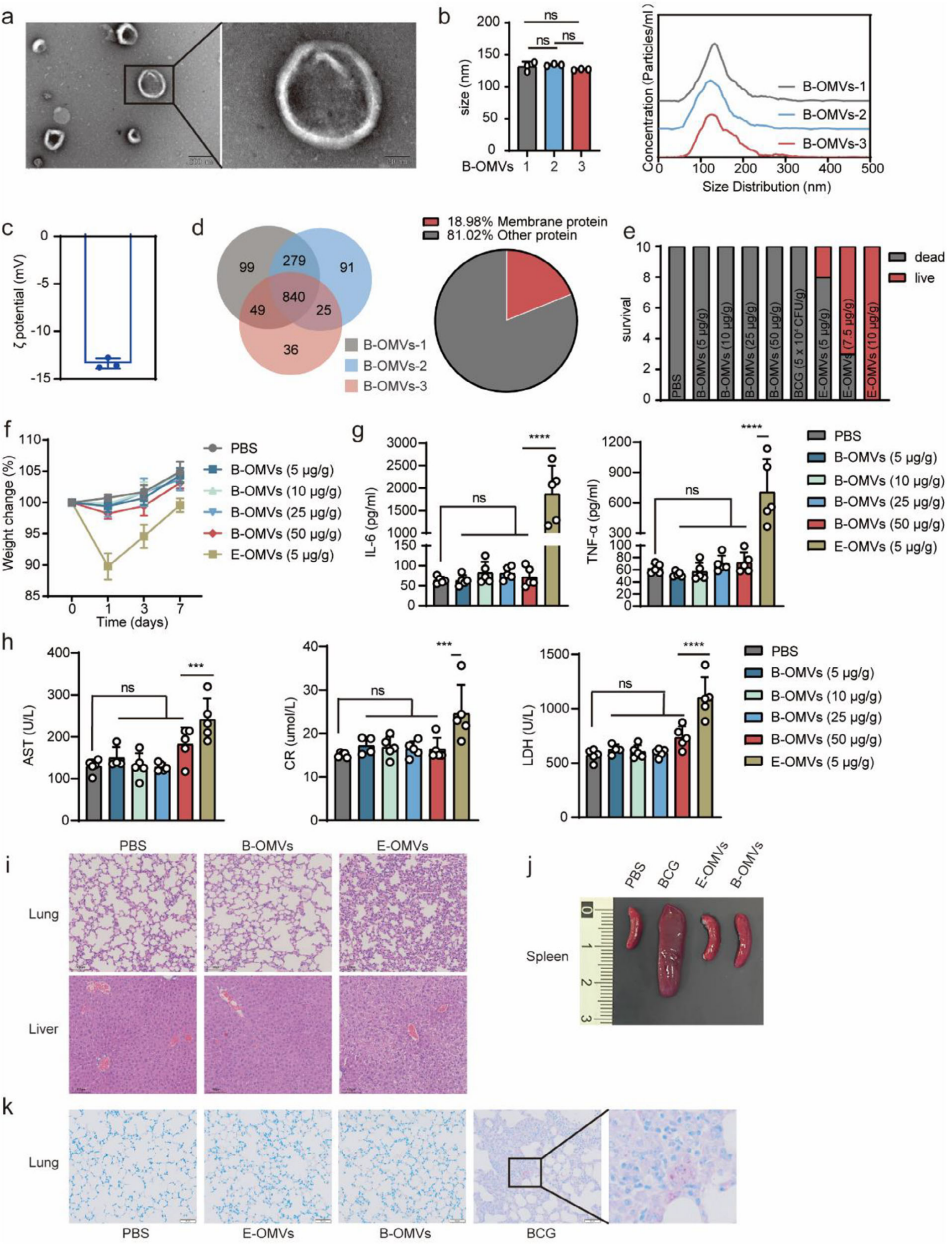
Characterization and safety evaluation of B‐OMVs.a) Representative TEM images of B‐OMVs. Scale bar: 200 nm (left panel); 50 nm (right panel).b) Nanoparticle Tracking Analysis results of B‐OMVs.c) Theζ‐potential of B‐OMVs.d) Proteomic sequencing analysis of B‐OMVs.e) Survival rate of mice after treatment with the indicated doses of B‐OMVs, BCG, or E‐OMVs (n = 10 mice per group). f) Body weights of mice treated with the indicated doses of B‐OMVs, BCG, or E‐OMVs (n = 5 mice per group).g) Serum levels of IL‐6 and TNF‐𝛼 in mice 24 h after treatment with the indicated doses of B‐OMVs, BCG or E‐OMVs (n = 5 mice per group).h) Blood biochemistry analysis of AST, CR, and LDH levels in the serum of mice 24 h after treatment with the indicated doses of B‐OMVs, BCG, or E‐OMVs (n = 4‐5 mice per group).i) H&E staining of lung and liver tissues 24 h after treatment with PBS (control), B‐OMVs (50 µg g^−1^), E‐OMVs (5 µg g^−1^) and BCG (5 × 10^4^ CFU g^−1^). Scale bar: 100 µm. j) Representative images of spleen 21 days after treatment with PBS (control), B‐OMVs (50 µg g^−1^), E‐OMVs (5 µg g^−1^) and BCG (5 × 10^4^ CFU g^−1^). Scale bar: 100 µm.k) Acid‐fast bacilli (AFB) staining of lung tissues of mice from j. Scale bar: 50 µm.Data are presented as means ± SD. Statistical significance was analyzed using one‐way ANOVA with Tukey's multiple comparisons test (b) and one‐way ANOVA with Dunnett's multiple comparisons test (g and h). ns, not significant; *p < 0.05, **p < 0.01, ***p < 0.001, and ****p < 0.001.

We isolated and collected outer membrane vesicles derived from BCG, and B‐OMVs, and subsequently characterized their morphology and composition. The transmission electron microscopy (TEM) revealed that BCG‐OMVs were spherical in shape, with a clearly defined outer membrane (**Figure** **1**a). To quantify their size, we used nanoparticle tracking analysis, which confirmed that B‐OMVs were biological nanoparticles with an average size of ≈130 nm (Figure 1b). In addition, the ζ‐potential of B‐OMVs was measured as [−13.37 ± 0.55] mV (mean ± SD) (Figure 1c).

To further characterize the composition of B‐OMVs, we conducted proteomic sequencing analysis. The protein expression profiles of B‐OMVs across different batches exhibited minimal variation, suggesting consistent production (Figure 1d; Figure S1a, Supporting Information). A significant proportion of the identified proteins were membrane proteins (19%), including lipoproteins such as MPT83, LpqN/LpqT, LprG, and LppX/LprAFG, indicating the vesicles' membrane‐centric composition (Figure 1d; Figure S1a,b, Supporting Information). Moreover, SDS‐PAGE analysis compared the protein banding patterns of B‐OMVs with those of BCG, *E. coli*‐derived OMVs (E‐OMVs), and whole *E. coli*, revealing distinct profiles among these preparations (Figure S1c, Supporting Information).

### 2.2 B‐OMVs Exhibit Minimal Toxicity, Surpassing BCG and E‐OMVs in Safety

We next assessed the cytotoxicity of B‐OMVs compared with E‐OMVs. Endotoxin levels were quantified using the Limulus amebocyte lysate (LAL) assay, revealing that B‐OMVs were LPS‐free, whereas E‐OMVs exhibited high endotoxin content (Figure S1d, Supporting Information). To assess cellular toxicity, bone marrow‐derived macrophages (BMDMs), a key immune cell type in trained immunity, were treated with varying doses of B‐OMVs or E‐OMVs, and cell viability was measured using the CCK‐8 assay. At low doses, no significant difference in cytotoxicity was observed between B‐OMVs and E‐OMVs. However, high doses of B‐OMVs demonstrated reduced cytotoxicity compared to E‐OMVs (p<0.001) (Figure S1e, Supporting Information). Hemolytic activity analysis revealed that both B‐OMVs and E‐OMVs caused less than 5% hemolysis at all doses, with B‐OMVs at the highest dose (100 µg mL^−1^) causing less hemolysis than E‐OMVs (p<0.0001) (Figure S1f, Supporting Information). These results suggest that B‐OMVs offer a broader therapeutic dose range with minimal cytotoxicity compared to E‐OMVs.

To further assess the safety of B‐OMVs in vivo, we intraperitoneally injected 6‐8‐week‐old mice with diverse doses of B‐OMVs, and E‐OMVs. At doses of 5, 7.5, and 10 µg g^−1^, E‐OMVs caused dose‐dependent mortality, with 100% mortality at 10 µg g^−1^. In contrast, all mice survived following B‐OMV treatment, even at the highest dose of 50 µg g^−1^ (Figure 1e). The lowest dose of E‐OMVs (5 µg g^−1^), which had minimal impact on survival, was used for subsequent experiments. No significant body weight changes were observed in the B‐OMVs‐treated groups, while mice treated with E‐OMVs at 5 µg g^−1^ exhibited significant weight loss (Figure 1f). Serum analysis on day 1 showed significantly lower levels of inflammatory cytokines interleukin 6 (IL‐6) and tumor necrosis factor alpha (TNF‐α) (Figure 1g) and markers of tissue damage (aspartate aminotransferase [AST], creatinine [CR], and lactate dehydrogenase [LDH]) in the B‐OMVs group compared to E‐OMVs (p<0.001), with no significant differences between B‐OMVs and PBS controls (Figure 1h). Histological analysis of lung and liver tissues showed that E‐OMVs induced tissue damage and inflammatory cell infiltration, while B‐OMVs treatment showed no significant changes, similar to the PBS control (Figure 1i).

Given that the clinical application of BCG is limited by its reactogenicity, including adverse effects such as BCGitis and disseminated BCGosis, we further investigated whether B‐OMVs could circumvent these complications. Gross examination revealed splenomegaly in some BCG‐treated mice (Figure 1j), and acid‐fast bacilli staining of lung tissues showed chronic inflammation and granuloma formation, indicating pulmonary BCG infection, which did not occur in B‐OMVs‐treated mice (Figure 1k).

Overall, B‐OMVs treatment demonstrated no significant cytotoxicity or pathological effects compared to E‐OMVs or BCG.

### 2.3 B‐OMVs Induce Better Trained Immunity and Protect Mice Against Microbial Sepsis

**Figure 2:**
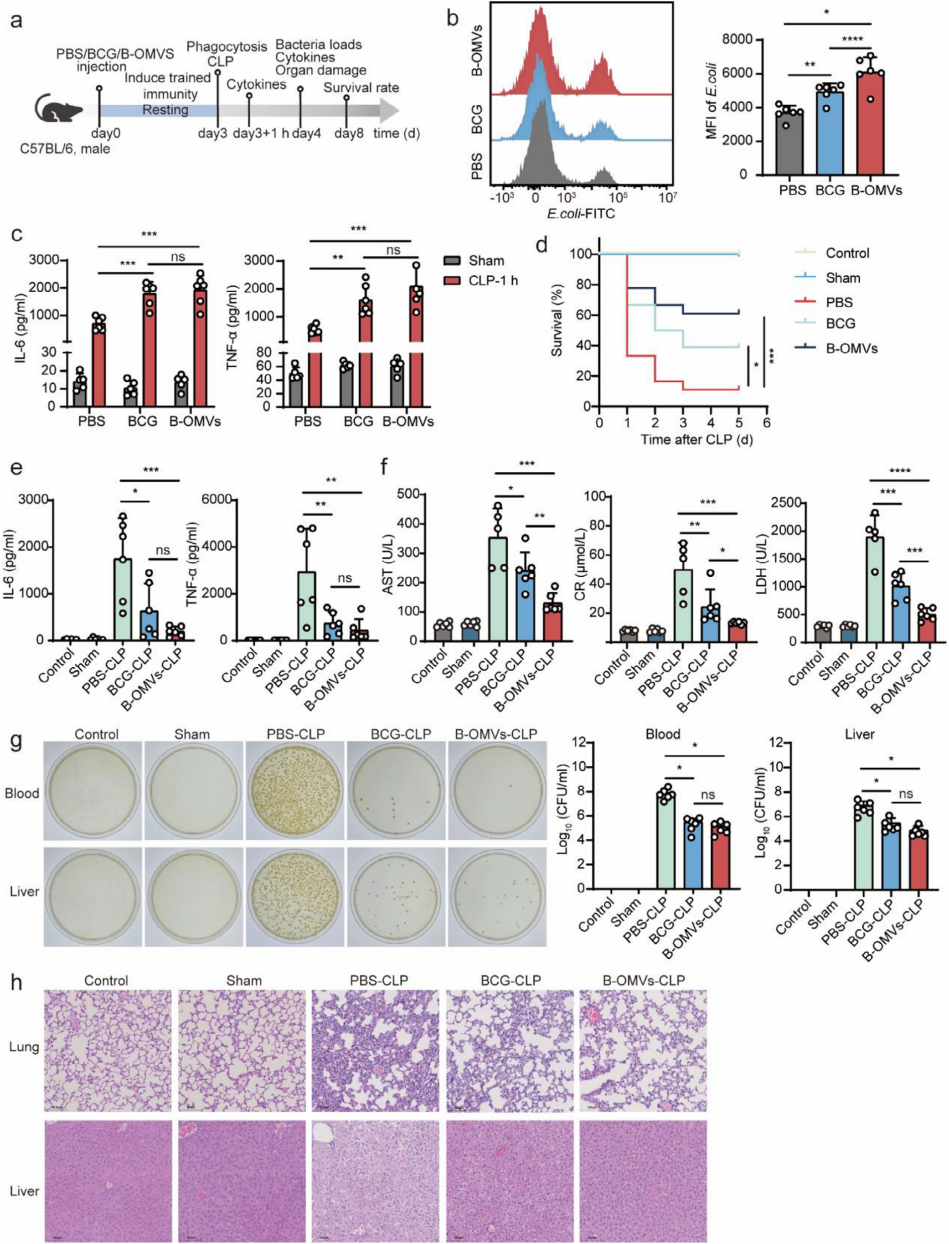
B‐OMVs induce trained immunity and protect mice against microbial sepsis.a) Schematic of B‐OMVs‐induced trained immunity and subsequent assays. 6‐8‐week‐old mice were treated with PBS (control), BCG (5 × 10^4^ CFU g^−1^), or B‐OMVs (5 µg g^−1^). After a 3‐day resting period, the mice underwent CLP to induce sepsis. b) Phagocytosis assay of peripheral blood from the indicated groups. Peripheral blood was collected 3 days after PBS (control), BCG (5 × 10^4^ CFU g^−1^), or B‐OMVs (5 µg g^−1^) injection and incubated with green fluorescent‐labeled *E. coli* for 20 min. Phagocytosis was assessed by flow cytometry and the MFI were quantified (right panel) (n = 6 mice per group). c) Serum levels of IL‐6 and TNF‐𝛼 in mice from the indicated groups 1 h after CLP (n = 6 mice per group).d) Survival rate of mice in the indicated groups after CLP (n = 18 mice per group).e) Serum levels of IL‐6 and TNF‐𝛼 in mice from the indicated groups 24 h after CLP (n = 6 mice per group).f) Blood biochemistry analysis of AST, CR, and LDH levels in serum of mice from the indicated groups 24 h after CLP (n = 5‐6 mice per group).g) Bacterial loads of blood and liver from the indicated groups 24 h after CLP. Representative plates were shown (left panel). Bacterial colonies were quantified (right panel) (n = 6 mice per group).h) H&E staining of lung and liver tissues from the indicated groups 24 h after CLP. Scale bar: 100 µm.Data are presented as means ± SD. Statistical significance was analyzed using one‐way ANOVA with Dunnett's multiple comparisons test (b, c, e, f, and g) and Log‐rank (Mantel‐Cox) test (d). ns, not significant; *p < 0.05, **p < 0.01, ***p < 0.001, and ****p < 0.001.

To evaluate whether B‐OMVs could induce trained immunity and protect against microbial sepsis, 6‐8‐week‐old mice were treated with PBS (control), BCG, or B‐OMVs. After a 3‐day resting period, mice underwent cecal ligation and puncture (CLP) to induce polymicrobial sepsis (**Figure** **2**a).

To determine the optimal doses of BCG and B‐OMVs, mice were administered varying concentrations of each agent. Three days later, CLP was performed. Survival was monitored, and serum samples were collected 24 h post‐CLP to assess systemic cytokine levels (IL‐6 and TNF‐α) and tissue injury markers (AST, CR, and LDH). Among the tested doses, 5 × 10⁴ CFU/g of BCG and 5 µg/g of B‐OMVs yielded the most favorable outcomes, including significantly improved survival and reduced levels of pro‐inflammatory cytokines and tissue damage. Therefore, these doses were selected for subsequent experiments (Figure S2a,d, Supporting Information). To assess the ability of B‐OMVs‐trained immune cells to initiate phagocytosis and inflammatory responses, peripheral blood was collected 3 days post‐training and incubated with green fluorescent‐labeled, heat‐inactivated *E. coli*. Flow cytometry revealed that B‐OMVs treatment enhanced leukocyte phagocytosis (Figure 2b). Proinflammatory cytokine assays at 1 h post‐septic challenge showed that B‐OMVs induced significantly higher levels of IL‐6 and TNF‐α compared to PBS treatment. Although cytokine levels were not significantly different from those induced by BCG, B‐OMVs consistently exhibited a higher trend, suggesting a potentially stronger proinflammatory response in the early stages of sepsis (Figure 2c).

Survival monitoring indicated that BCG treatment improved survival from 11.11% in PBS‐treated mice to 38.89% (p = 0.0275). Remarkably, B‐OMVs treatment conferred the greatest protection, with survival exceeding 60% (p = 0.001) (Figure 2d). To further assess whether B‐OMVs‐induced trained immunity enhanced tissue protection and antimicrobial responses, we collected serum and organs 24 h post‐septic challenge to evaluate systemic cytokine levels, tissue damage markers, and bacterial clearance.

B‐OMVs treatment reduced serum IL‐6 and TNF‐α levels compared to PBS and BCG groups (p<0.05) (Figure 2e). Consistently, B‐OMVs‐treated mice exhibited the lowest levels of tissue damage markers AST, CR, and LDH compared to PBS and BCG groups (p<0.05) (Figure 2f). B‐OMVs treatment also reduced bacterial loads in the blood and visceral organs (liver, spleen, lungs, heart, and kidneys) compared to both PBS and BCG treatment groups, indicating enhanced bacterial clearance (Figure 2g; Figure S2e,f, Supporting Information). Histological analysis of lung and liver tissues by H&E staining showed that B‐OMVs treatment mitigated tissue damage and inflammatory cell infiltration following septic challenge, compared to both PBS and BCG groups (Figure 2h).

These findings demonstrate that B‐OMVs‐induced trained immunity activates a rapid immune response and enhances antimicrobial activity, thereby facilitating efficient pathogen clearance and mitigating the risk of cytokine storms and multi‐organ dysfunction in the context of sepsis.

### 2.4 B‐OMVs Induce Trained Immunity by Triggering HSC Expansion and Enhancing Myelopoiesis

**Figure 3:**
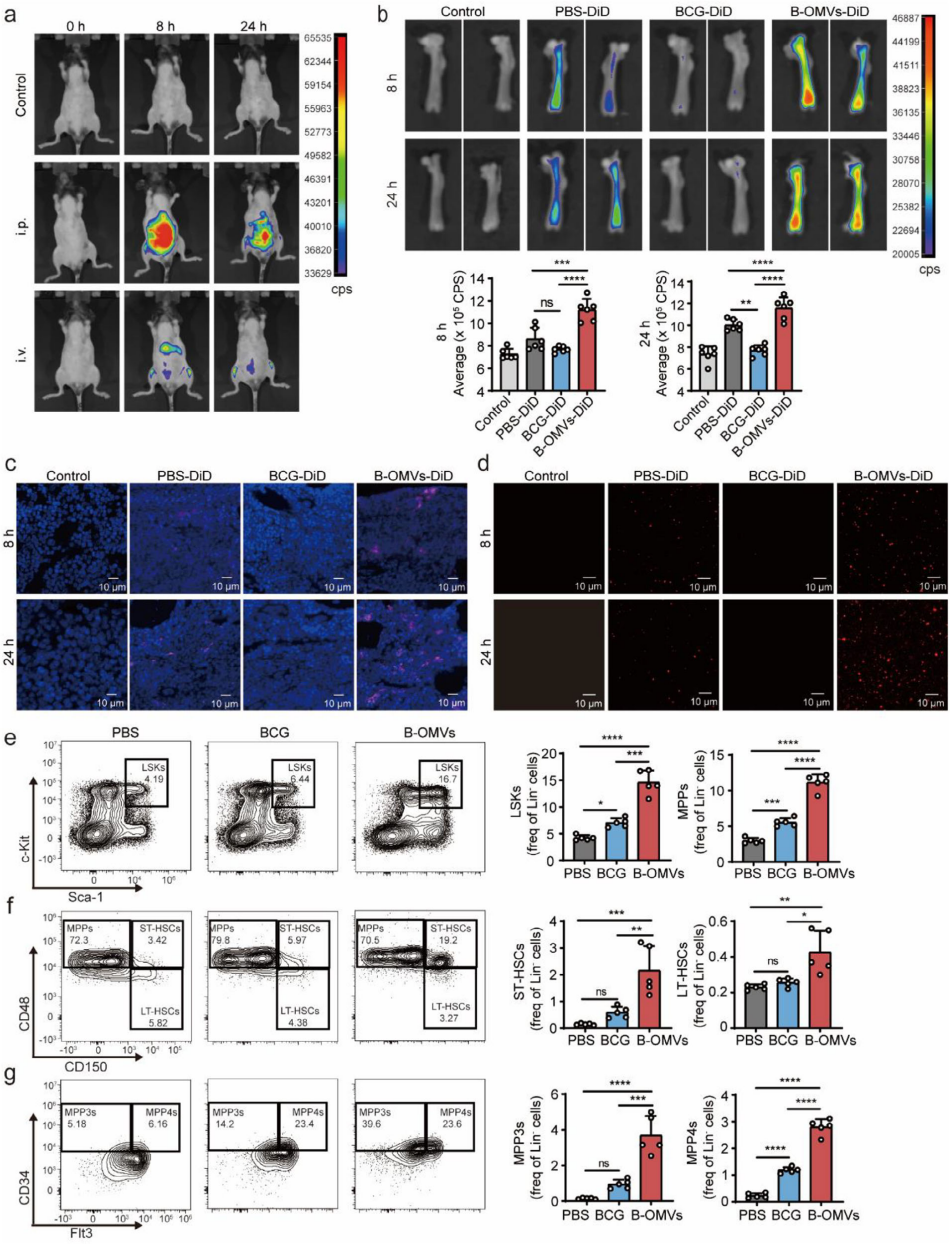
B‐OMVs trigger HSCs expansion and enhance myelopoiesis.a) The imaging of mice injected intraperitoneally or intravenously with DiD‐labeled B‐OMVs (5 µg g^−1^) for 8 and 24 h. b) The *ex vivo* imaging of femurs from mice injected intraperitoneally with PBS (control), DiD‐labeled BCG (5 × 10^4^ cfu g^−1^), and B‐OMVs (5 µg g^−1^) for 8 and 24 h. Fluorescence intensity was quantified (low panel) (n = 6 mice per group).c) Immunofluorescence staining of femur sections from mice injected intraperitoneally with PBS (control), DiD‐labeled BCG (5 × 10^4^ cfu/g), and B‐OMVs (5 µg g^−1^) for 8 and 24 h. Scale bar: 10 µm.d) Fluorescence microscopy images of bone marrow cells isolated from mice injected intraperitoneally with PBS (control), DiD‐labeled BCG (5 × 10^4^ cfu g^−1^), and B‐OMVs (5 µg g^−1^) for 8 and 24 h. Scale bar: 10 µm.e‐g) 6‐8‐week‐old mice were treated with PBS (control), BCG (5 × 10^4^ cfu g^−1^), or B‐OMVs (5 µg g^−1^). After a 3‐day resting period, bone marrow cells were collected for flow cytometry analysis.e) Flow cytometry analysis of hematopoietic progenitors (LSKs) in bone marrow from the indicated groups. The frequencies were quantified (right panel) (n = 5 mice per group).f) Flow cytometry analysis of MPPs, ST‐HSCs, and LT‐HSC in bone marrow from the indicated groups. The frequencies were quantified (right panel) (n = 5 mice per group).g) Flow cytometry analysis of MPP3s and MPP4s in bone marrow from the indicated groups. The frequencies were quantified (right panel) (n = 5 mice per group).Data are presented as means ± SD. Statistical significance was analyzed using one‐way ANOVA with Dunnett's multiple comparisons test (b, e, f, and g). ns, not significant; *p < 0.05, **p < 0.01, ***p < 0.001, and ****p < 0.001.

To investigate the mechanisms underlying B‐OMVs‐induced trained immunity, we administered DiD‐labeled B‐OMVs intraperitoneally (i.p.) or intravenously (i.v.) to 6–8‐week‐old mice. Using a small animal imager, we observed the distribution of B‐OMVs to the lower limbs at both 8‐ and 24‐h post injection, regardless of the administration route (**Figure** **3**a). Subsequently, femurs were harvested, and imaging confirmed an accumulation of B‐OMVs in the bone marrow, while no such accumulation was detected for BCG (Figure 3b). This finding aligns with previous reports indicating that BCG does not directly stimulate bone marrow cells to induce trained immunity but instead relies on NOD‐like receptor 2 (NOD2) signaling.^[^ ^22^, ^41^ ^]^ Moreover, size‐matched liposomes did not exhibit comparable bone marrow accumulation, suggesting that the tropism of B‐OMVs cannot be attributed solely to their nanoscale size (Figure S3a, Supporting Information). Further immunofluorescence staining of femur sections revealed red fluorescently labeled B‐OMVs in the bone marrow (Figure 3c). Consistent with these results, fluorescence microscopy of isolated bone marrow cells confirmed the localization of B‐OMVs (Figure 3d).

Trained immunity is known to involve the modulation of hematopoietic stem and progenitor cells (HSPCs) in the bone marrow and their myeloid progeny.^[^ ^13^, ^42^, ^43^ ^]^ To examine changes in HSPCs following B‐OMV administration, we injected 6–8‐week‐old mice intraperitoneally with B‐OMVs. After a 3‐day resting period, bone marrow cells were collected for flow cytometry analysis (gating strategies are shown in Figure S3b, Supporting Information). Compared to PBS and BCG treatment groups, B‐OMVs treatment significantly increased the frequencies of hematopoietic progenitors (LSKs, Lin⁻c‐kit⁺sca‐1⁺) and multipotent progenitors (MPPs, CD48⁺CD150⁻ LSKs) (Figure 3e).

Notably, B‐OMVs treatment resulted in a marked increase in short‐term hematopoietic stem cells (ST‐HSCs, CD150⁺CD48⁺ LSKs), with up to a 10‐fold increase compared to the PBS group. Long‐term HSCs (LT‐HSCs, CD150⁺CD48⁻ LSKs) also showed a modest increase (Figure 3f). B‐OMVs treatment preferentially induced myeloid‐biased progenitors (MPP3s, CD150⁻CD48⁺CD34⁺Flt3⁻ LSKs), but not lymphoid‐biased progenitors (MPP4s, CD150⁻CD48⁺CD34⁺Flt3⁺ LSKs), compared to BCG treatment (Figure 3g).

To further assess whether B‐OMVs affect mature myeloid cells in the periphery, flow cytometry was performed to analyze monocytes and neutrophils in peripheral blood. Compared to the PBS group, both B‐OMVs and BCG treatments increased the proportion of myeloid leukopoiesis (CD11b⁺) (Figure S3c, Supporting Information), with a significant rise in neutrophils (CD11b⁺Ly6C⁻Ly6G⁺), but no changes in monocytes (CD11b⁺Ly6C⁺Ly6G⁻) (Figure S3d, Supporting Information). We also analyzed dendritic cell (DC) populations in both bone marrow and peripheral blood, and observed no significant differences across treatment groups (Figure S3e,f, Supporting Information), indicating that B‐OMVs do not markedly affect DC abundance.

**Figure 4:**
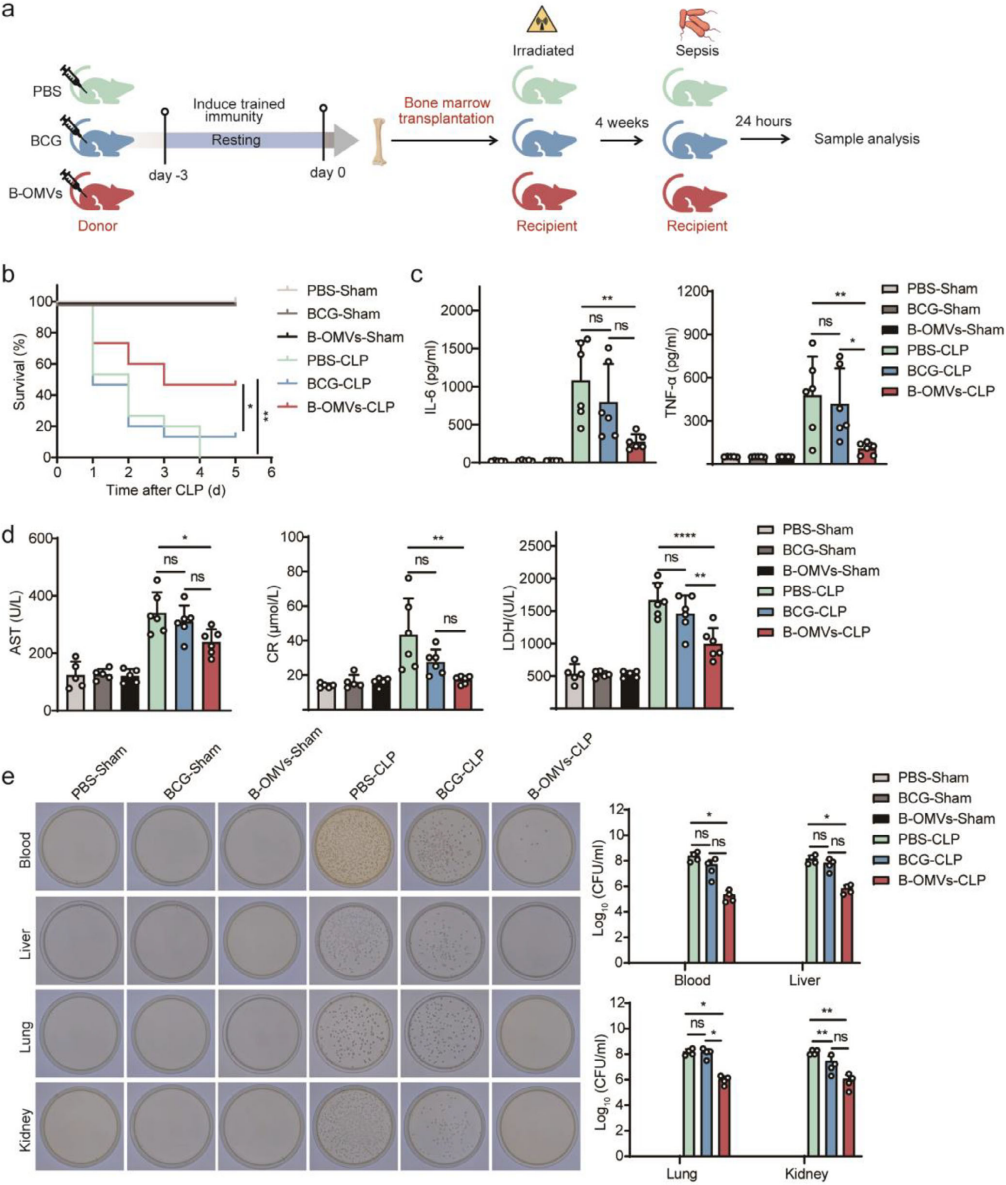
B‐OMVs induce central trained immunity.a) Schematic illustration of the bone marrow transplantation experiment and subsequent analyses. Donor mice were treated intraperitoneally with PBS, BCG (5 × 10⁴ CFU g^−1^), or B‐OMVs (5 µg g^−1^). Three days later, bone marrow cells were harvested and intravenously transferred (5 × 10⁶ cells per mouse) into lethally irradiated recipient mice (896 cGy). After four weeks of hematopoietic reconstitution, recipient mice were subjected to CLP‐induced sepsis.b) Survival curves of recipient mice in the indicated groups after CLP (sham: n = 8 mice per group; CLP: n = 15 mice per group).c) Serum levels of IL‐6 and TNF‐𝛼 in mice from the indicated groups 24 h after CLP (n = 5‐6 mice per group).d) Blood biochemistry analysis of AST, CR, and LDH levels in serum of mice from the indicated groups 24 h after CLP (n = 5‐6 mice per group).e) Bacterial loads of blood, liver, lung and kidney from the indicated groups 24 h after CLP (n = 4 mice per group).Data are presented as means ± SD. Statistical significance was analyzed using one‐way ANOVA with Dunnett's multiple comparisons test (c‐e) and Log‐rank (Mantel‐Cox) test (b). ns, not significant; *p < 0.05, **p < 0.01, ***p < 0.001, and ****p < 0.001.

To directly assess the induction of central trained immunity by B‐OMVs, we performed bone marrow transplantation experiments. Donor mice were treated with PBS (control), BCG (5 × 10⁴ CFU/g), or B‐OMVs (5 µg g^−1^), and bone marrow cells were harvested three days later for intravenous transplantation into lethally irradiated recipient mice. After four weeks of hematopoietic reconstitution, recipient mice were subjected to CLP to induce sepsis (**Figure** **4**a). Recipients of bone marrow from B‐OMVs‐treated donors exhibited significantly improved survival, reduced systemic cytokine levels, attenuated tissue injury, and enhanced bacterial clearance in blood and major organs compared to recipients of PBS‐ or BCG‐derived marrow (Figure 4b–e).

To further distinguish central from peripheral contributions to trained immunity, we conducted parabiosis experiments. Mice pretreated with PBS, BCG, or B‐OMVs were surgically conjoined to untreated partners three days post‐treatment to establish shared circulation. After two weeks, the untreated parabionts were subjected to CLP (denoted as P‐CLP) (Figure S4a, Supporting Information). No significant differences in survival, inflammatory cytokine production, tissue injury, or bacterial burden were observed among P‐CLP mice paired with B‐OMVs‐, BCG‐, or PBS‐treated partners (Figure S4b–e, Supporting Information), indicating that circulating immune factors or trained peripheral cells alone are insufficient to confer protection.

Collectively, these findings demonstrate that B‐OMVs confer protection against sepsis primarily through the central programming of bone marrow myeloid progenitors. Mechanistically, B‐OMVs promote the expansion of HSCs and their differentiation toward myeloid‐biased progenitors, thereby enhancing innate immune memory and host defense.

### 2.5 Trained Immunity Induced by B‐OMVs Augments the Inflammatory Response and Phagocytosis of BMDMs In Vitro

To evaluate whether B‐OMVs‐induced changes in HSCs and MPPs could affect the function of bone marrow‐derived monocytes/macrophages (Mo/Macs), we investigated the induction of trained immunity by B‐OMVs in vitro using BMDMs. Trained immunity is driven by the recognition of PAMPs and danger‐associated molecular patterns (DAMPs) through PRRs.^[^ ^13^, ^44^ ^]^ To assess the interaction between BMDMs and B‐OMVs, we co‐cultured DiO‐labeled B‐OMVs (green) with Dil‐labeled BMDMs (red) and examined their co‐localization using laser confocal microscopy. As shown in Figure S5a (Supporting Information), B‐OMVs were efficiently internalized by BMDMs, as evidenced by distinct co‐localization. Proinflammatory cytokine assays revealed that B‐OMVs treatment increased the secretion of IL‐6 and TNF‐α from BMDMs compared to both PBS and BCG treatment groups (Figure S5b, Supporting Information), indicating the potent immunomodulatory effects of B‐OMVs.

**Figure 5:**
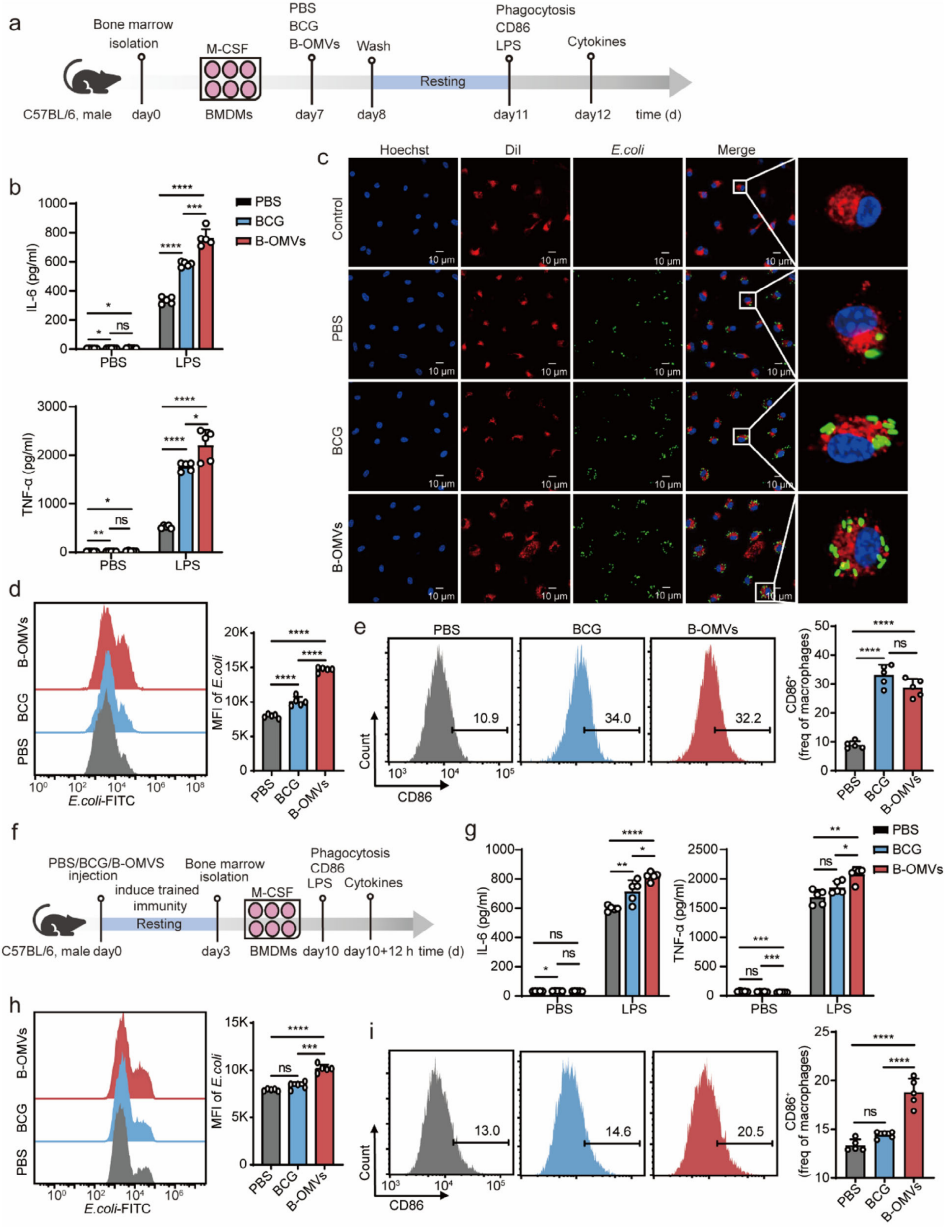
Trained immunity induced by B‐OMVs enhances inflammatory response and phagocytosis of BMDMs.a) Schematic of B‐OMVs‐induced trained immunity in vitro and subsequent assays. BMDMs were treated with PBS (control), BCG (1 × 10^4^ CFU ml^−1^), or B‐OMVs (5 µg ml^−1^) for 24 h. After a 3‐day resting period, the cells were stimulated by LPS (100 ng ml^−1^) for 12 h.b) Levels of IL‐6 and TNF‐𝛼 in the supernatant from the indicated groups with or without LPS stimulation (n = 5).c‐d) Phagocytosis assay of BMDMs from the indicated groups. BMDMs were collected 3 days after PBS (control), BCG (1 × 10^4^ CFU ml^−1^), or B‐OMVs (5 µg ml^−1^) training and incubated with green fluorescent‐labeled *E. coli* for 20 min. Phagocytosis was assessed by laser confocal microscopy (c) and flow cytometry (d) (n = 5). Scale bar: 10 µm.e) Flow cytometry analysis of CD86^+^ cell proportions in CD11b^+^ F4/80^+^ cells from the indicated groups. BMDMs were collected 3 days after PBS (control), BCG (1 × 10^4^ CFU ml^−1^), or B‐OMVs (5 µg ml^−1^) training and analyzed by Flow cytometry. The proportion of CD86⁺ cells in CD11b⁺F4/80⁺ population was quantified (right panel) (n = 5).f) Schematic of B‐OMVs‐induced trained immunity in vivo and stimulation in vitro. 6‐8‐week‐old mice were treated with PBS (control), BCG (5 × 10^4^ CFU g^−1^), or B‐OMVs (5 µg g^−1^). After a 3‐day resting period, bone marrow cells were isolated and stimulated by M‐CSF for 7 days to differentiate into BMDMs in vitro. BMDMs were stimulated by LPS (100 ng ml^−1^) for 12 h. g) Levels of IL‐6 and TNF‐𝛼 in the supernatant from the indicated groups with or without LPS stimulation (n = 5).h) Phagocytosis assay of BMDMs from the indicated groups. BMDMs were collected and incubated with green fluorescent‐labeled *E. coli* for 20 min. Phagocytosis was assessed by flow cytometry and the MFI were quantified (right panel) (n = 5).i) Flow cytometry analysis of CD86^+^ cell proportions in CD11b^+^ F4/80^+^ cells from the indicated groups. BMDMs were collected and analyzed by Flow cytometry. The proportion of CD86⁺ cells in CD11b⁺F4/80⁺ population was quantified (right panel) (n = 5).Data are presented as means ± SD. Statistical significance was analyzed using one‐way ANOVA with Dunnett's multiple comparisons tests (b, d, e, g, h, and i). ns, not significant; *p < 0.05, **p < 0.01, ***p < 0.001, and ****p < 0.001.

To determine whether B‐OMVs induce trained immunity and enhance inflammatory response and phagocytosis of BMDMs, we treated BMDMs with PBS (control), BCG, or B‐OMVs for 24 h (**Figure** **5**a). After a three‐day resting period, BMDMs were further stimulated with LPS, and proinflammatory cytokine levels of IL‐6 and TNF‐α in the supernatant were quantified. Upon LPS stimulation, B‐OMVs‐treated BMDMs exhibited higher levels of IL‐6 and TNF‐α than PBS‐ and BCG‐treated BMDMs (*p* < 0.05) (Figure 5b).

We then assessed the phagocytic capacity of BMDMs by co‐culturing them with fluorescently labeled *E. coli*. B‐OMVs‐treated BMDMs displayed enhanced bacterial phagocytosis compared to PBS‐ and BCG‐treated BMDMs, as confirmed by laser confocal microscopy (Figure 5c) and FACScan analysis (Figure 5d). Trained immunity also promotes macrophage polarization toward the M1 phenotype (pro‐inflammatory).^[^ ^45^ ^]^ FACScan analysis revealed an increased M1 macrophage population following both BCG and B‐OMVs treatments (Figure 5e, gating strategies are shown in Figure S5c, Supporting Information).

To validate these findings in vivo, we induced trained immunity in mice, extracted bone marrow cells, and differentiated them into BMDMs in vitro (Figure 5f). Consistent with in vitro results, BMDMs trained with B‐OMVs exhibited enhanced inflammatory responses, phagocytosis, and M1 macrophage polarization (p<0.05 vs PBS and BCG treatment groups) (Figure 5g–i). In contrast, BCG‐induced trained immunity resulted in a moderate inflammatory response but had no effects on phagocytosis or M1 macrophage polarization (Figure 5g–i), underscoring the superior potency and durability of B‐OMVs‐induced trained immunity.

### 2.6 Metabolic Characteristics of Trained Immunity Induced by B‐OMVs

**Figure 6:**
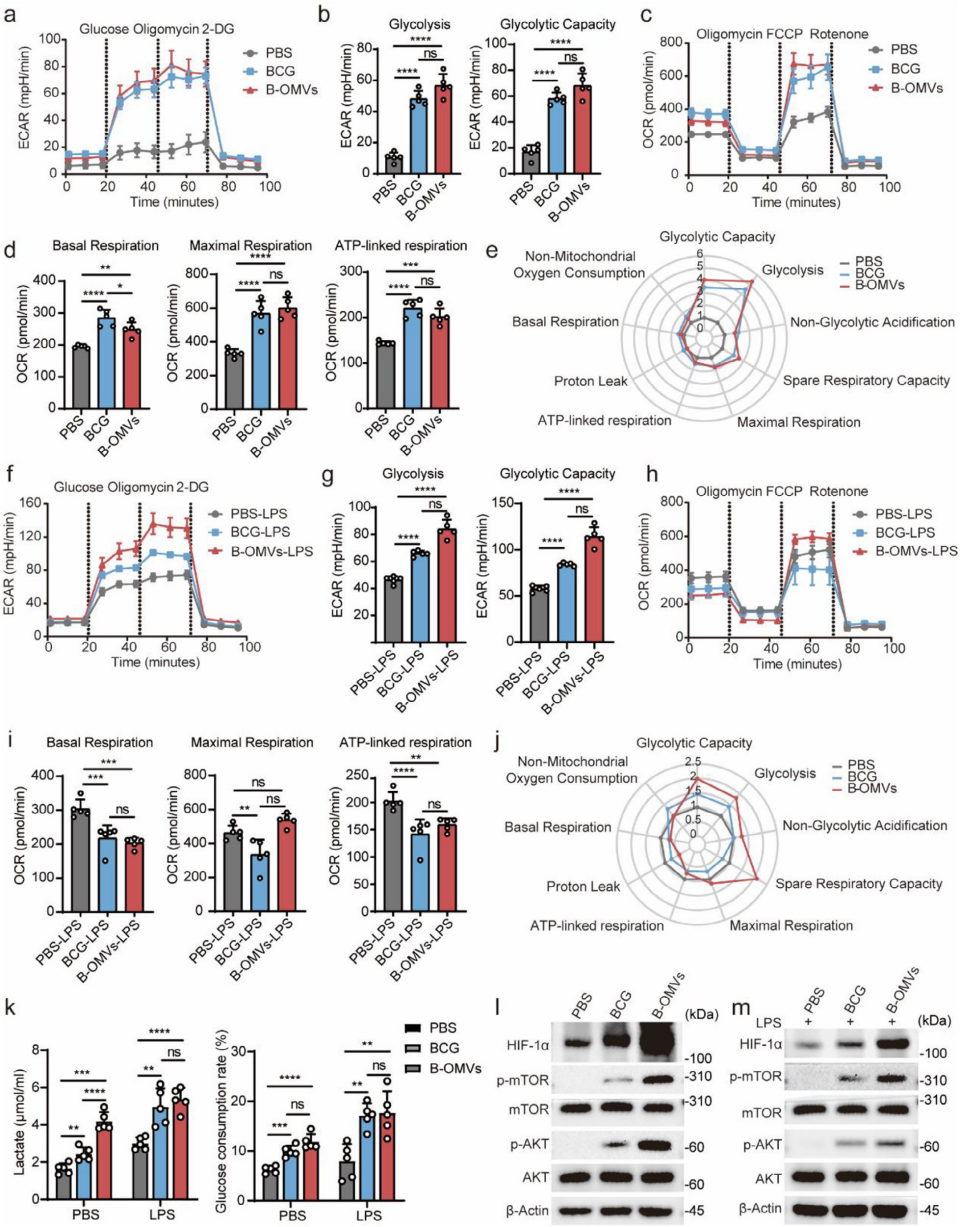
Trained immunity induced by B‐OMVs is associated with increased glycolytic metabolism.a‐e) BMDMs were treated with PBS (control), BCG (1 × 10^4^ CFU ml^−1^), and B‐OMVs (5 µg ml^−1^) for 24 h, followed by a 3‐day resting period. Seahorse XF technology was used to evaluate metabolic changes in aerobic glycolysis and OXPHOS.a) Seahorse analysis of glycolytic metabolism in BMDMs from the indicated groups.b) The levels of glycolysis and glycolytic capacity of BMDMs from the indicated groups (n = 5). c) Seahorse analysis of mitochondrial OXPHOS in BMDMs from the indicated groups.d) The levels of basal respiration, maximal respiration, and ATP‐linked respiration of BMDMs from the indicated groups (n = 5).e) Radar plot analysis of aerobic glycolysis and mitochondrial OXPHOS in BMDMs from the indicated groups.f‐k) BMDMs were treated with PBS (control), BCG (1 × 10^4^ CFU ml^−1^), and B‐OMVs (5 µg ml^−1^) for 24 h. After a 3‐day resting period, the cells were stimulated by LPS (100 ng ml^−1^) for 12 h. Seahorse XF technology was used to evaluate metabolic changes in aerobic glycolysis and OXPHOS (f‐j). The supernatant was collected to measure the levels of lactate and glucose (k). f) Seahorse analysis of glycolytic metabolism in BMDMs from the indicated groups after LPS stimulation.g) The levels of glycolysis and glycolytic capacity of BMDMs from the indicated groups after LPS stimulation (n = 5). h) Seahorse analysis of mitochondrial OXPHOS in BMDMs from the indicated groups after LPS stimulation.i) The levels of basal respiration, maximal respiration, and ATP‐linked respiration of BMDMs from the indicated groups after LPS stimulation (n = 5).j) Radar plot analysis of aerobic glycolysis and mitochondrial OXPHOS in BMDMs from the indicated groups after LPS stimulation. k) The levels of lactate and glucose in the supernatant of BMDMs from the indicated groups after LPS stimulation (n = 5).l) Western blot analysis of proteins involved in pathways of the Akt‐mTOR‐HIF axis in BMDMs from the indicated groups.m) Western blot analysis of proteins involved in pathways of the Akt‐mTOR‐HIF axis in BMDMs from the indicated groups after LPS stimulation.ECAR, extracellular acidification rate; OCR, oxygen consumption rate; 2‐DG, 2‐deoxy‐D‐glucose. Data are presented as means ± SD. Statistical significance was analyzed using one‐way ANOVA with Dunnett's multiple comparisons tests (b, d, g, i, and k). ns, not significant; *p < 0.05, **p < 0.01, ***p < 0.001, and ****p < 0.001.

Metabolic reprogramming is a critical feature of trained immunity, characterized by significant upregulation of aerobic glycolysis and oxidative phosphorylation (OXPHOS).^[^ ^46^ ^]^ To examine the metabolic phenotype of BMDMs trained with B‐OMVs, BMDMs were treated with PBS, BCG, or B‐OMVs for 24 h, followed by a 3‐day resting period. Seahorse XF technology was used to evaluate metabolic changes in aerobic glycolysis and OXPHOS. The results demonstrated that BCG‐ and B‐OMVs‐induced trained immunity significantly enhanced both glycolysis (*p* < 0.0001) (**Figure** **6**a,b) and OXPHOS (*p* < 0.01) (Figure 6c,d) compared to the PBS control. Notably, glycolytic parameters (glycolysis, glycolytic capacity, and non‐glycolytic acidification) exhibited more substantial changes than OXPHOS parameters (basal respiration, maximal respiration, ATP‐linked respiration, spare respiratory capacity, proton leak, and non‐mitochondrial oxygen consumption) (Figure 6e; Figure S6a,d, Supporting Information).

Immune cells undergo glycolytic reprogramming, a metabolic shift from OXPHOS to glycolysis, to effectively mount a rapid immune response to antigenic stimulation.^[^ ^47^ ^]^ To further explore the metabolic phenotype after secondary stimulation in B‐OMVs‐induced trained immunity, BMDMs were stimulated with LPS for 12 h. Upon LPS stimulation, glycolysis levels increased across all groups, with higher levels observed in the BCG‐ and B‐OMVs‐trained groups compared to the PBS control (*p* < 0.01) (Figure 6f,g; Figure S6e, Supporting Information). OXPHOS exhibited opposite alterations, namely both BCG‐ and B‐OMVs‐trained groups showed reduced OXPHOS levels compared to the PBS group (*p* < 0.05) (Figure 6h). Specific OXPHOS parameters, including basal respiration, maximal respiration, and ATP‐linked respiration, were lower in the BCG‐ and B‐OMVs‐trained groups than in the PBS group (Figure 6i; Figure S6f–h, Supporting Information). Radar plot analysis corroborated these findings (Figure 6j). These results suggest that while B‐OMVs‐induced trained immunity enhances both glycolysis and OXPHOS, the immune response to a secondary stimulation is primarily driven by glycolysis. In trained immunity, elevated glycolytic activity leads to increased lactate production and glucose consumption.^[^ ^48^ ^]^ To confirm this, lactate and glucose levels in the supernatant of BMDMs were measured. BMDMs treated with BCG or B‐OMVs exhibited higher lactate accumulation and glucose consumption compared to the PBS control, a trend that persisted after LPS stimulation (Figure 6k), further supporting the activation of aerobic glycolysis in B‐OMVs‐induced trained immunity.

The AKT‐ the mechanistic target of rapamycin (mTOR)‐hypoxia‐inducible factor 1‐alpha (HIF‐1α) pathway plays a pivotal role in metabolic reprogramming of trained immunity by inducing aerobic glycolysis.^[^ ^48^ ^]^ To investigate the regulatory mechanisms underlying B‐OMVs‐induced glycolytic metabolism, we analyzed key signaling proteins. Western blot analysis revealed that B‐OMVs treatment increased the expression of phosphorylated AKT (p‐AKT), phosphorylated mTOR (p‐mTOR), and HIF‐1α compared to PBS and BCG treatment groups (Figure 6l). Furthermore, upon LPS stimulation, these proteins remained at significantly elevated levels in the B‐OMVs‐treated group (Figure 6m), suggesting that B‐OMVs‐induced trained immunity enhances aerobic glycolysis through activation of the AKT‐mTOR‐HIF‐1α pathway.

### 2.7 Epigenetic Characteristics of Trained Immunity Induced by B‐OMVs

Trained immunity is characterized by two key processes: epigenetic and metabolic reprogramming.^[^ ^46^ ^]^ Its induction, maintenance, and regulation depend on the interplay between metabolic pathways and epigenetic machinery. After observing that B‐OMVs‐induced trained immunity enhances aerobic glycolysis, we further investigated whether this process is driven by epigenetic reprogramming using transposase‐accessible chromatin sequencing (ATAC‐seq) to analyze chromosome structure in BMDMs.

**Figure 7:**
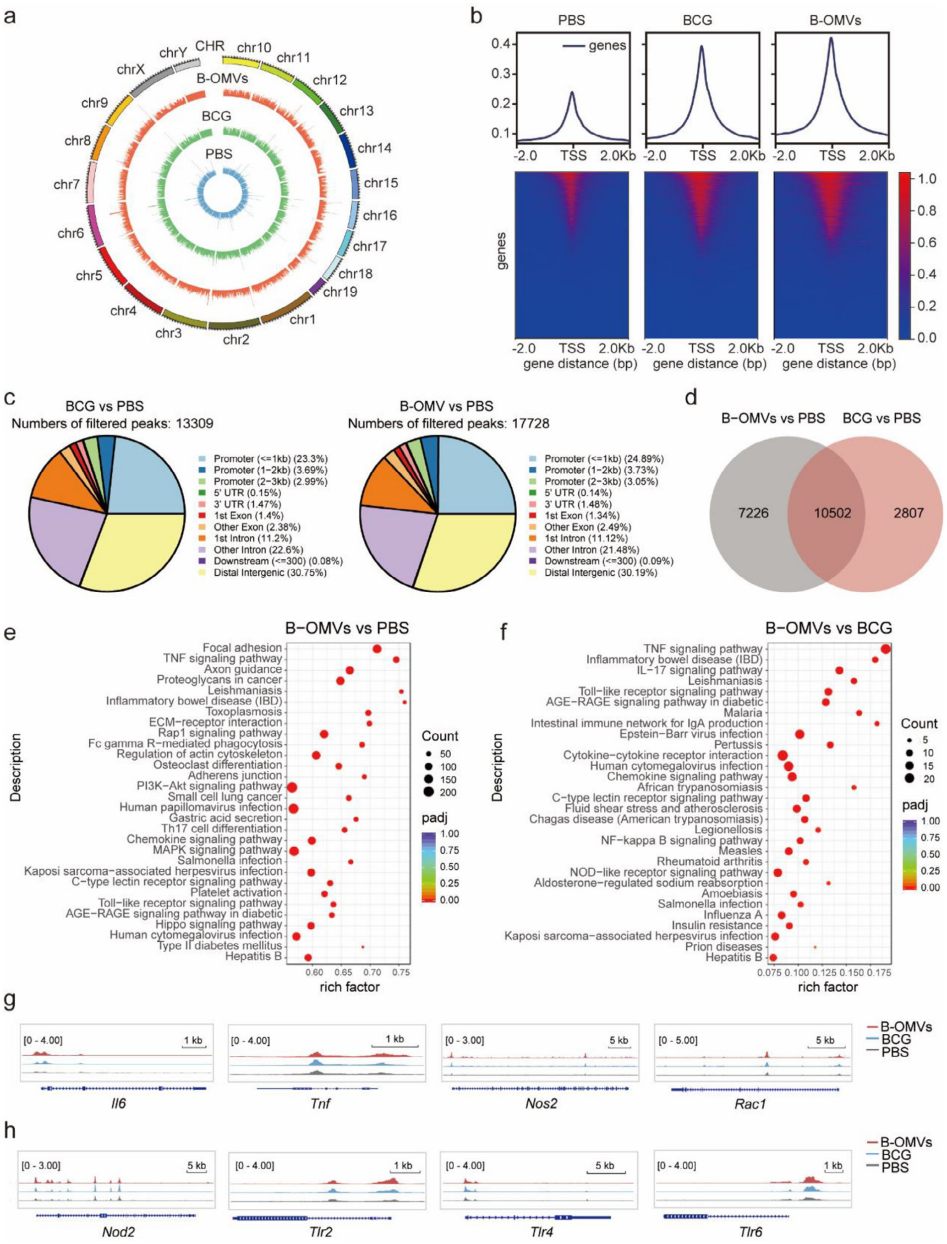
B‐OMVs modulate the epigenetic landscape of BMDMs.a) Genome‐wide chromatin accessibility of BMDMs treated with PBS (control), BCG (1 × 10^4^ CFU ml^−1^), and B‐OMVs (5 µg ml^−1^). b) Heatmaps of the differential assay for ATAC peak (P < 0.05) densities at TSS regions of BMDMs treated with PBS (control), BCG (1 × 10^4^ CFU ml^−1^), and B‐OMVs (5 µg ml^−1^). bp, base pairs. c) Differential ATAC peak distributions on gene loci. UTR, untranslated region. d) Venn diagram analysis of genes with differential ATAC peaks.e) Heatmap comparison between PBS‐treated group and B‐OMVs‐treated groups. KEGG enrichment analysis of genes with differential ATAC peaks. f) Heatmap comparison between BCG‐treated group and B‐OMVs‐treated groups. KEGG enrichment analysis of genes with differential ATAC peaks.g) ATAC signals of pro‐inflammatory genes (*Il6* and *Tnf*) and phagocytosis‐associated genes (*Rac1* and *Nos2*). h) ATAC signals of pattern recognition receptor genes (*Nod2*, *Tlr2*, *Tlr4*, and *Tlr6*).

We identified 21,146, 42,250, and 39,253 open chromatin regions (OCRs) in the PBS, BCG, and B‐OMVs groups, respectively. Compared with the PBS group, chromatin accessibility was increased in both BCG and B‐OMVs groups, with the highest levels observed in the B‐OMVs group (**Figure** **7**a). Consistently, the highest ATAC signals on transcription start sites (TSSs) were detected in genes from BMDMs treated with B‐OMVs (Figure 7b). Comparative analysis revealed 13,309 and 17,728 differential peaks in the BCG and B‐OMVs groups, respectively, compared with the PBS group. These peaks were mainly distributed in promoter, distal intergenic regions, and introns (Figure 7c). Venn diagram analysis of these differential peaks demonstrated 10,502 overlapping peaks between the BCG and B‐OMVs groups, along with 2,807 and 7,226 unique peaks in the BCG and B‐OMVs groups, respectively (Figure 7d). These results indicate that while BCG and B‐OMVs share common features of trained immunity, distinct differences also exist. KEGG enrichment analysis of genes with differential ATAC peaks (GAPs) revealed that immune signaling pathways, particularly proinflammatory signaling pathways, were significantly enriched in both BCG and B‐OMVs groups (Figure 7e; Figure S7a, Supporting Information). The B‐OMVs group displayed greater enrichment in pattern recognition receptor signaling pathways, including the Toll‐like receptor signaling pathway (Figure 7f). By using Integrative Genomics Viewer (IGV), we further observed increased chromatin accessibility in genes involved in pathways of the Akt‐mTOR‐HIF axis (*Hif1a*, *Mtor*, *Pik3ca*, *Rps6*, and *Rps6ka2*) and glycolysis (*Hk2*, *Ldha*, *Pdk1*, *Pfkp*, *Pgam1*, *Pgk1*, *Pmk*, and *Slc2a1*) after training compared with the control (Figure S7b,c, Supporting Information). Similarly, proinflammatory genes (*Il6* and *Tnf*) and phagocytosis‐associated genes (*Rac1* and *Nos2*) also showed increased chromatin accessibility (Figure 7g). This was more pronounced in the B‐OMVs group than in the BCG group. Furthermore, the B‐OMVs group displayed enhanced chromatin accessibility in pattern recognition receptor genes, particularly *Tlr2*, compared with the PBS and BCG groups, likely driven by interactions between bacterial lipoproteins in B‐OMVs and TLR2 (Figure 7h). To validate the observed epigenetic changes at the transcriptional level, we performed RNA‐seq analysis. Consistent with the ATAC‐seq results, genes involved in glycolysis, inflammation, and innate immune activation were significantly upregulated in the B‐OMVs group, supporting the notion that B‐OMVs induce transcriptionally active epigenetic reprogramming (Figure S7d, Supporting Information).

### 2.8 B‐OMVs Induce Trained Immunity in a TLR2‐Dependent Manner

PRRs, particularly TLRs such as TLR2, TLR4, and TLR9, are critical for recognizing *Mycobacterium tuberculosis* (Mtb).^[^ ^49^ ^]^ BCG, derived from Mtb, naturally releases OMVs containing TLR2 lipoprotein agonists, which interact with mouse macrophages to regulate immune responses via a TLR2‐dependent mechanism.^[^ ^50^ ^]^ Based on ATAC‐seq analysis, we hypothesize that B‐OMVs induce trained immunity through TLR2.

**Figure 8:**
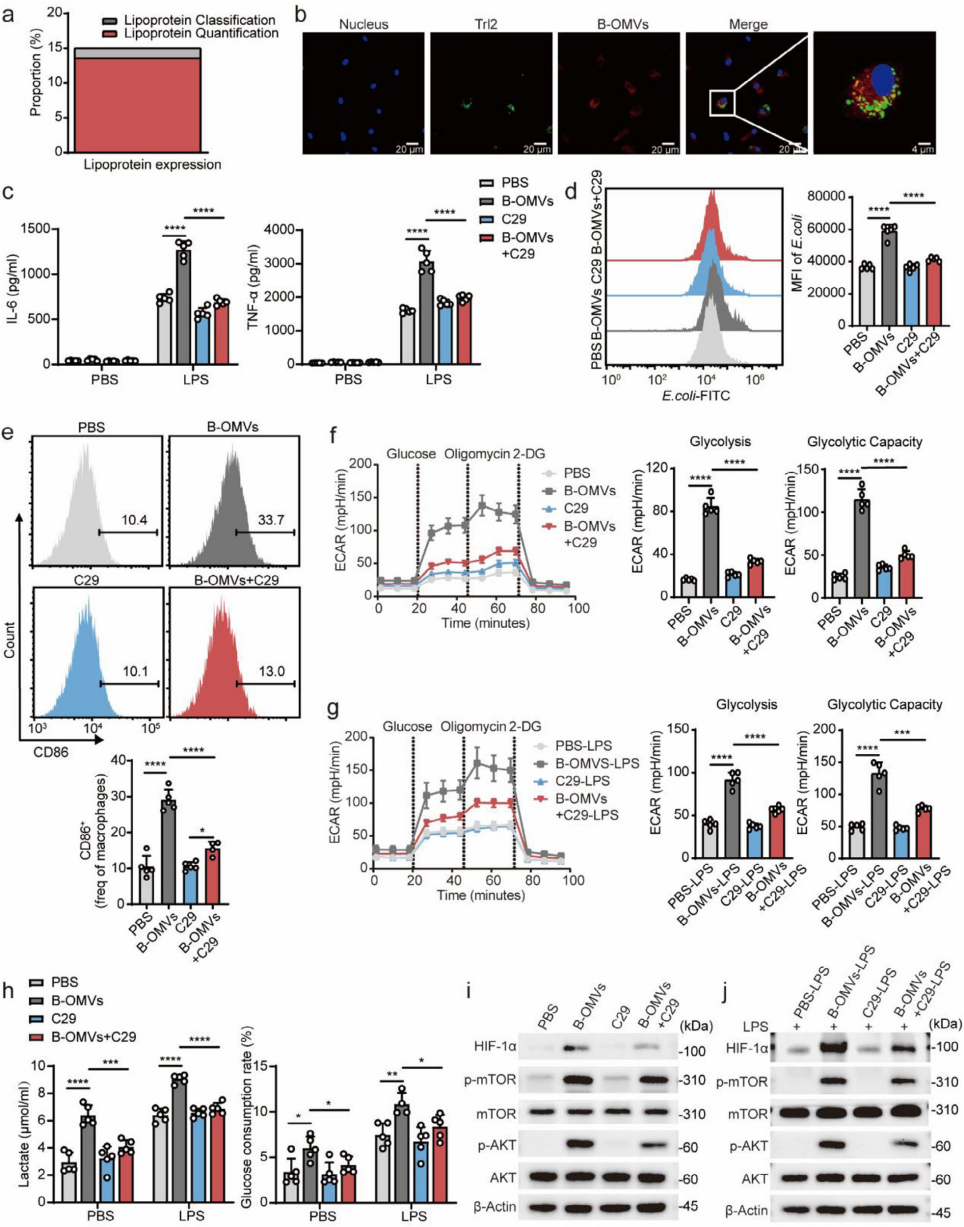
B‐OMVs induce trained immunity in a TLR2 dependent manner.a) The analysis of protein composition in B‐OMVs.b) Immunofluorescence analysis of Tlr2 and B‐OMVs. BMDMs were treated with 5 µg ml^−1^ Dil‐labeled B‐OMVs (red) for 2 h and co‐localization was examined by laser confocal microscopy. Scale bar: 20 µm.c‐j) BMDMs were treated with PBS (control), B‐OMVs (5 µg ml^−1^), C29 (100 µm) as well as B‐OMVs (5 µg ml^−1^) and C29 (100 µm) for 24 h. After a 3‐day resting period, the cells were stimulated by LPS (100 ng ml^−1^) for 12 h. c) Levels of IL‐6 and TNF‐𝛼 in the supernatant from the indicated groups with or without LPS stimulation (n = 5).d) Phagocytosis assay of BMDMs from the indicated groups. BMDMs were collected and incubated with green fluorescent‐labeled *E. coli* for 20 min. Phagocytosis was assessed by flow cytometry and the MFI was quantified (right panel) (n = 5).e) Flow cytometry analysis of CD86^+^ cell proportions in CD11b^+^ F4/80^+^ cells from the indicated groups. BMDMs were collected and analyzed by Flow cytometry. The proportion of CD86⁺ cells in CD11b⁺F4/80⁺ population was quantified (right panel) (n = 5).f) Seahorse analysis of glycolytic metabolism in BMDMs from the indicated groups. The levels of glycolysis and glycolytic capacity were quantified (right panel) (n = 5).g) Seahorse analysis of glycolytic metabolism in BMDMs from the indicated groups after LPS stimulation. The levels of glycolysis and glycolytic capacity were quantified (right panel) (n = 5).h) The levels of lactate and glucose in the supernatant of BMDMs from the indicated groups with or without LPS stimulation (n = 5).i) Western blot analysis of proteins involved in pathways of the Akt‐mTOR‐HIF axis in BMDMs from the indicated groups.j) Western blot analysis of proteins involved in pathways of the Akt‐mTOR‐HIF axis in BMDMs from the indicated groups after LPS stimulation.ECAR, extracellular acidification rate; 2‐DG, 2‐deoxy‐D‐glucose. Data are presented as means ± SD. Statistical significance was analyzed using one‐way ANOVA with Dunnett's multiple comparisons test (c‐h). ns, not significant; *p < 0.05, **p < 0.01, ***p < 0.001, and ****p < 0.001.

To test this hypothesis, we analyzed proteomic sequencing data from three different batches of B‐OMVs. Among the 840 shared proteins identified, 12 were bacterial lipoproteins, representing 1.4% of the total protein categories. However, these lipoproteins accounted for 13.6% of the total protein abundance, indicating their significant enrichment within B‐OMVs (**Figure** **8**a). To evaluate whether B‐OMVs interact with TLR2, we performed immunofluorescence analysis, which revealed the clear colocalization of B‐OMVs with TLR2 (Figure 8b). C29, a widely used TLR2 inhibitor, competitively blocks the initiation of TLR2 dimerization by binding to its extracellular domain, thereby inhibiting TLR2 activation.^[^ ^51^ ^]^ Subsequently, we inhibited TLR2 activation using C29 to examine the role of TLR2 in B‐OMV‐induced trained immunity. As shown in Figure 8c, C29 treatment attenuated the release of proinflammatory cytokines IL‐6 and TNF‐α upon LPS stimulation. Consistently, FACScan analysis demonstrated that C29 suppressed B‐OMVs‐induced enhancement of phagocytosis and M1 proinflammatory macrophage polarization (Figure 8d,e).

To further investigate the role of TLR2 in B‐OMVs‐induced metabolic reprogramming, we conducted Seahorse XF assays. C29 treatment significantly attenuated B‐OMVs‐enhanced glycolysis levels, regardless of LPS stimulation (Figure 8f,g; Figure S8a, Supporting Information). Similarly, C29 treatment reduced B‐OMVs‐induced lactate accumulation and increased glucose consumption both before and after LPS stimulation (Figure 8h; Figure S8b, Supporting Information). Since TLR2 is upstream of the AKT‐mTOR‐HIF‐1α pathway, its inhibition is expected to suppress downstream signaling. Western blot analysis confirmed that p‐AKT, p‐mTOR, and HIF‐1α levels were reduced in the C29‐treated B‐OMVs group compared to the B‐OMVs‐only group (Figure 8i). Furthermore, these levels remained lower in the C29‐treated B‐OMVs group upon LPS stimulation (Figure 8j), suggesting that B‐OMVs induce trained immunity via a TLR2‐dependent mechanism.

## 3 Discussion

Despite significant advancements in sepsis management, the long‐term prognosis for survivors remains a major challenge, with ≈40% of patients re‐hospitalized within 90 days due to recurrent infections associated with sepsis‐induced immunosuppression.^[^ ^33^, ^52^, ^53^ ^]^ This persistent immune dysfunction underscores the need for therapeutic strategies that not only address acute sepsis but also mitigate its long‐term effects on the immune system. Current immunomodulatory therapies—such as granulocyte‐macrophage colony‐stimulating factor (GM‐CSF),^[^ ^54^, ^55^, ^56^ ^]^ interferon‐gamma (IFN‐γ),^[^ ^57^, ^58^, ^59^ ^]^ interleukin‐7 (IL‐7),^[^ ^60^, ^61^ ^]^ immune checkpoint inhibitors,^[^ ^62^ ^]^ and intravenous immunoglobulin^[^ ^63^, ^64^, ^65^ ^]^—are being investigated to restore immune homeostasis in sepsis. However, safety concerns and potential toxicity, especially associated with extracellular vesicles derived from pathogenic microorganisms^[^ ^66^ ^]^ or mesenchymal stromal cells,^[^ ^67^ ^]^ highlight the urgent need for more effective and safer therapeutic strategies that can boost host defenses without inducing harmful side effects.

In this study, we developed B‐OMVs as a novel immunomodulatory agent and demonstrated their robust protective effects against microbial sepsis in a murine CLP model of polymicrobial sepsis. B‐OMVs induced trained immunity, enhanced innate immune function, and significantly improved survival. These findings support B‐OMVs as a promising immunotherapeutic strategy with potential applications in sepsis vaccination and the broader treatment of infectious diseases.

The fundamental feature of trained immunity is the long‐lasting enhancement of innate immune responses, facilitated by epigenetic reprogramming and metabolic changes in innate immune cells such as monocytes, macrophages, and neutrophils.^[^ ^13^, ^14^ ^]^ HSCs are crucial to this process, as they respond to infection by rapidly proliferating and differentiating into myeloid progenitors to replenish the immune system. In addition to this immediate response, HSCs undergo epigenetic modifications that enhance their capacity for more effective immune responses to subsequent infections. This process is a hallmark of trained immunity and contributes to a heightened immune defense against secondary infections.^[^ ^68^ ^]^ Previous studies have shown that agents such as β‐glucan^[^ ^42^ ^]^ or BCG vaccination^[^ ^19^ ^]^ promote the expansion of myeloid progenitors and elicit trained immunity. In line with these findings, our study confirms that B‐OMVs stimulate HSC expansion and differentiation into myeloid‐lineage progenitors, facilitating the induction of trained immunity. Importantly, B‐OMVs interact with the bone marrow, likely altering the bone marrow microenvironment, to prime HSCs. This contrasts with BCG, which does not directly engage with HSCs but instead binds to NOD2 receptors on other cells, such as monocytes or mesenchymal stem cells, within the HSC niche.^[^ ^69^, ^70^, ^71^, ^72^ ^]^

Our findings further reveal that B‐OMVs induce trained immunity in a TLR2‐dependent manner, distinguishing it from BCG‐induced immunity. Notably, treatment with C29, a TLR2 inhibitor, partially suppressed B‐OMVs‐induced trained immunity, suggesting that other signaling pathways, such as those mediated by C‐type lectin receptors and NOD‐like receptors, may also play a role. Our ATAC‐seq analysis supports this hypothesis by indicating additional pathways involved in B‐OMVs‐induced trained immunity. These results highlight the broader immunomodulatory potential of B‐OMVs, which may account for the stronger and more sustained immune response observed compared to BCG treatment.

BCG, a live attenuated strain of Mycobacterium bovis, is the only WHO‐recommended vaccine for tuberculosis and has been used globally since its introduction in 1921.^[^ ^73^, ^74^, ^75^ ^]^ In addition to its primary role in tuberculosis prevention, BCG is known to induce trained immunity, which has led to its exploration for other therapeutic applications.^[^ ^74^ ^]^ However, BCG's use is limited by its safety profile, particularly in immunocompromised individuals, where it can cause serious adverse effects such as BCGitis or disseminated BCG disease.^[^ ^76^, ^77^, ^78^, ^79^ ^]^ These limitations highlight the need for safer alternatives to BCG that retain its immunomodulatory effects but reduce the risk of adverse events. In this study, we explored B‐OMVs as a safer alternative to BCG and demonstrated that, unlike BCG, B‐OMVs are non‐replicative, and exhibit no significant cytotoxicity or pathological effects. Furthermore, B‐OMVs elicited a stronger trained immunity response than BCG, supporting their potential as a more effective and safer immunomodulatory strategy.

Despite the promising results of this study, several limitations warrant further investigation. Most notably, the use of murine models poses inherent challenges for translating findings to human sepsis.^[^ ^80^ ^]^ While mice are valuable for elucidating pathophysiological mechanisms and testing therapeutic strategies, interspecies differences—particularly in immune cell populations,^[^ ^81^, ^82^ ^]^ and glucose metabolism^[^ ^83^ ^]^ —may generalizability of the results. For example, neutrophils exhibit considerable phenotypic heterogeneity across tissues and inflammatory states in both humans and mice, highlighting the need for caution when extrapolating murine data to human disease.^[^ ^82^ ^]^ To overcome these limitations, future studies should incorporate non‐human primate models or advanced humanized mouse systems that more closely mimic human immune responses and disease pathology. Ultimately, clinical trials are essential to evaluate the safety and efficacy of B‐OMVs in humans, with data from randomized controlled studies serving as a critical foundation for future translational applications.

In conclusion, we demonstrate that B‐OMVs are a safe and effective inducer of trained immunity, providing robust protection against microbial sepsis. By promoting HSC expansion and myelopoiesis through TLR2‐dependent activation of aerobic glycolysis and epigenetic reprogramming, B‐OMVs enhance inflammatory responses and phagocytosis in bone marrow‐derived macrophages. These findings position B‐OMVs as a promising immunomodulatory agent with superior safety and efficacy compared to conventional BCG vaccines and other extracellular vesicles. B‐OMVs represent a promising therapeutic strategy for managing sepsis and other infectious diseases, but rigorous clinical trials are needed to validate their safety and efficacy in human patients.

## 4 Materials and Methods

### Reagents

The sources of key reagents and materials are detailed in Table S1 (Supporting Information).

### Bacterial Culture

BCG was resuspended in 500 µL of sterile PBS (Servicebio) and plated onto Middlebrook 7H10 agar (Solarbio) supplemented with 10% OADC (Solarbio). The plates were then incubated at 37 °C for 14 days. A single colony was subsequently inoculated into 10 mL of Middlebrook 7H9 Broth Base (Solarbio) containing 10% ADC (Solarbio), 0.05% Tween‐80 (Solarbio), and 0.2% glycerol (Solarbio), and cultured at 37 °C with shaking at 250 rpm for 14 days. Following this, 1 mL of the bacterial suspension was transferred into 500 mL of Middlebrook 7H9 medium and incubated at 37 °C with shaking at 250 rpm until the OD600 reached 0.6–0.8 (logarithmic growth phase). The bacterial suspension was used for subsequent experiments, while the remaining culture was stored in a 25% glycerol solution at ‐80 °C for future use. E. coli was cultured in LB liquid medium at 37 °C with shaking.

### Mice

All experiments conducted in this study were reviewed and approved by the Ethical Committee of Soochow University (Approval No. SUDA 20231228A01) and were performed in accordance with the Guide for the Care and Use of Laboratory Animals, as published by the US National Institutes of Health (NIH Publication No. 85‐23, revised 1996). Male C57BL/6 mice, aged 6 to 8 weeks, were obtained from JOINN Laboratories (Suzhou, China) and were maintained in a specific pathogen‐free (SPF) environment.

### Isolation of BMDMs

Bone marrow cells were extracted from the femurs and tibiae of 6‐ to 8‐week‐old male C57BL/6 mice. The harvested bone marrow was cultured in DMEM (Gibco) supplemented with 20% heat‐inactivated fetal bovine serum (Gibco), 100 units mL^−1^ penicillin, 100 µg mL^−1^ streptomycin sulfate, and 20 ng/mL recombinant mouse macrophage colony‐stimulating factor (M‐CSF, Stemcell). On day 3, a fresh culture medium was added. On day 5, the original medium was replaced with fresh complete DMEM containing 10% heat‐inactivated fetal bovine serum (Gibco), 100 units mL^−1^ penicillin, and 100 µg mL^−1^ streptomycin sulfate. The cells were cultured until day 7, at which point mature BMDMs were obtained.

### Isolation of BCG‐Derived OMVs

OMVs were collected as follows: 500 mL of bacterial culture medium was centrifuged at 3,450 g for 15 min at 4 °C to remove bacterial cells, followed by filtration through a 0.45 µm polyethersulfone filter (Millipore). The filtrate was concentrated 20‐fold using a 100 kDa ultrafiltration unit (Millipore) at 4,000 g. The concentrate was then ultracentrifuged at 100,000 g for 2 h at 4 °C (Beckman Coulter XPN‐100) to pellet the OMVs. The pellet was washed with 50 mL of PBS and ultracentrifuged again at 100,000 g for 2 h. The final OMV pellet was resuspended in PBS. In the following descriptions, OMVs isolated from BCG are collectively referred to as B‐OMVs. The concentration of B‐OMVs was determined using a Pierce BCA Protein Assay Kit (Thermo Scientific) and stored at ‐80 °C for subsequent experiments. OMVs derived from E. coli were isolated using a similar procedure.

### Characterization of B‐OMVs

A 10 µL drop of the B‐OMVs sample was applied to a carbon‐coated grid, which was then immediately negatively stained using uranyl acetate (phosphotungstic acid) for 1 min. The grids were examined using a JEM‐1400plus electron microscope operated at 120 kV. The particle size and concentration of B‐OMVs were measured using nanoparticle tracking analysis (NTA) with a Zetaview‐PMX120‐Z system (Particle Metrix, Meerbusch, Germany) and the corresponding ZetaView software (version 8.05.14 SP7). Isolated B‐OMVs samples were appropriately diluted in 1X PBS buffer for NTA measurements. The NTA analysis was recorded and performed at 11 positions. The ZetaView system was calibrated using 100 nm polystyrene particles, and the temperature was maintained between 23 °C and 30 °C.

### Proteomic Analysis of B‐OMVs

Ultra‐deep quantitative proteomic profiling of B‐OMVs was performed using the MetWare platform. Briefly, proteins were extracted from B‐OMVs using a lysis buffer (8 m urea, 1 mm PMSF, 2 mm EDTA) and disrupted by ultrasonic treatment on ice. The resulting protein mixture was then subjected to tryptic digestion into peptides. The tryptic peptide mixtures were analyzed using nano LC–MS/MS on a timsTOF Pro mass spectrometer (Bruker). Qualitative and quantitative protein analyses were carried out using DIA‐NN (v1.8.1) software.

### In Vivo Trained Immunity and Polymicrobial Sepsis

To investigate the role of trained immunity in polymicrobial sepsis, 6‐ to 8‐week‐old male C57BL/6 mice were intraperitoneally injected with PBS (control), BCG (5 × 10⁴ CFU/g body weight), or B‐OMVs (5 µg g^−1^ body weight) and allowed to rest for 3 days. Peripheral blood was collected under pentobarbital anesthesia, and the phagocytic activity of peripheral blood leukocytes was assessed using the pHrodo™ BioParticles™ Phagocytosis Kit (Invitrogen) to evaluate the enhanced phagocytic capacity conferred by trained immunity.

Polymicrobial sepsis was induced using the cecal ligation and puncture (CLP) model.^[^ ^84^, ^85^, ^86^ ^]^ Under pentobarbital anesthesia, mice were placed on a homeothermic blanket to maintain a body temperature of 36.5 °C. A small abdominal incision was made to expose the cecum, which was ligated below the ileocecal valve and punctured once with a 20‐gauge needle. The abdominal incision was then closed in layers. Sham‐operated mice underwent the same procedure without cecal ligation or puncture. Postoperative resuscitation was performed by subcutaneous injection of prewarmed normal saline (37 °C; 5 mL per 100 g body weight).

1 h after CLP, peripheral blood was collected under pentobarbital anesthesia to assess the early pro‐inflammatory response of trained immunity by measuring inflammatory cytokines in the blood. To evaluate the protective effects of trained immunity in sepsis, peripheral blood was collected again at 24 h post‐CLP under pentobarbital anesthesia. Mice were then euthanized by cervical dislocation, and organs, including the heart, liver, spleen, lungs, and kidneys, were harvested and homogenized for 1 min. The blood and organ homogenate suspensions were serially diluted and streaked onto LB agar plates for colony‐forming unit (CFU) counting. Liver and lung tissues were also collected for hematoxylin and eosin (H&E) staining.

Peripheral blood samples were stored at 4 °C overnight, followed by centrifugation at 2,500 rpm for 10 min to separate the serum, which was stored at ‐80 °C for subsequent analysis. For survival analysis, the survival status of mice was monitored daily for 5 consecutive days post‐CLP.

### In Vitro Trained Immunity

In the BMDM training model, BMDMs were seeded into flat‐bottom 48‐well cell culture plates (Corning) at a density of 2 × 10^5^ cells per well. To train the BMDMs, BCG (1 × 10^4^ CFU mL^−1^) and B‐OMVs (5 µg mL^−1^), or an equal volume of PBS (control group), were added to complete DMEM medium, and the cells were incubated at 37 °C for 24 h. After the training period, the supernatant was discarded, and the cells were washed three times with PBS. The medium was replaced with fresh complete DMEM, and the cells were rested for 3 days, with medium replacement as needed.

Phagocytic activity of trained BMDMs was evaluated using the pHrodo™ BioParticles™ Phagocytosis Kit (Invitrogen) or by co‐incubation with FITC‐labeled E. coli (bacteria:macrophage ratio = 20:1) for 20 min. Phagocytosis in the pHrodo™ BioParticles™ Phagocytosis Kit was analyzed by flow cytometry (Beckman Coulter), while FITC‐labeled E. coli phagocytosis was assessed using a laser scanning confocal microscope (Olympus).

To evaluate macrophage polarization, trained BMDMs were co‐incubated with anti‐CD11b‐PE (clone M1/70, BioLegend), anti‐F4/80‐FITC (clone BM8, BioLegend), and anti‐CD86‐PE‐Cy7 (clone GL1, eBioscience). Flow cytometry (Beckman Coulter) was used to detect polarization of BMDMs toward the M1 macrophage phenotype, defined as CD11b⁺ F4/80⁺ CD86^hi^ macrophages.

For cytokine release analysis, BMDMs were stimulated with *E. coli*‐derived lipopolysaccharide (LPS, 100 ng/mL; Sigma‐Aldrich) or an equal volume of PBS (control group) for 12 h. The supernatants were collected and stored at ‐80 °C for subsequent analysis.

### In Vivo Trained Immunity Followed by Ex Vivo Stimulation of BMDMs

Based on previously described methods for BMDM isolation and trained immunity models, 6–8‐week‐old male C57BL/6 mice were intraperitoneally injected with PBS (control), BCG (5 × 10^4^ CFU g^−1^ body weight), or B‐OMVs (5 µg g^−1^ body weight) and allowed to rest for 3 days. Bone marrow‐derived macrophages (BMDMs) were isolated from the femurs and tibiae of the mice and cultured for 10 days to obtain mature BMDMs.

On day 10, phagocytic capacity and polarization toward M1 macrophages (CD11b⁺ F4/80⁺ CD86^hi^) were assessed. Alternatively, the BMDMs were stimulated with *E. coli*‐derived lipopolysaccharide (LPS, 100 ng mL^−1^; Sigma–Aldrich) or an equal volume of PBS (control group) for 12 h. The supernatants were then collected and stored at ‐80 °C for subsequent cytokine analysis.

### Seahorse Experiments

The pretreatment of cells was based on the in vitro trained immunity method for BMDMs. Briefly, BMDMs were trained with BCG (1 × 10^4^ CFU mL^−1^) or B‐OMVs (5 µg mL^−1^) in complete DMEM, or an equivalent volume of PBS as a control, at 37 °C for 24 h. Following training, cells were washed three times with PBS, replaced with fresh medium, and rested for three days. Subsequently, the BMDMs were stimulated with *E. coli*‐derived LPS (100 ng mL^−1^; Sigma–Aldrich) or an equivalent volume of PBS as a control for 12 h. The trained BMDMs were then harvested and seeded at a density of 150,000 cells per well in a Seahorse XF24 microplate (Agilent Technologies). The cells were cultured overnight in complete DMEM at 37 °C and 5% CO₂ to allow for proper adherence.

After adherence, the complete DMEM was removed and replaced with Seahorse XF DMEM Base Medium (Agilent Technologies), supplemented according to the requirements of each metabolic assay. For the Glycolysis Stress Test, the medium was supplemented with 2 mm glutamine (Agilent Technologies). For the Cell Mito Stress Test, the medium was supplemented with 10 mm glucose, 1 mM pyruvate, and 2 mM glutamine (Agilent Technologies). The cells were then incubated in a CO₂‐free incubator at 37 °C for 60 min before the oxygen consumption rate (OCR) and extracellular acidification rate (ECAR) were measured at 37 °C using the Seahorse XF24 Extracellular Flux Analyzer (Agilent Technologies).

To evaluate the dynamic changes in the glycolytic rate, the Seahorse XF Glycolysis Stress Test was performed to monitor ECAR. During the test, cells were sequentially treated at specific time points (as indicated in Figure 5a and f) with 10 mm glucose (Agilent Technologies), 1 µm oligomycin (Agilent Technologies), and 50 mm 2‐deoxy‐D‐glucose (2‐DG; Agilent Technologies).

To assess the dynamic changes in mitochondrial oxygen consumption, the Seahorse XF Cell Mito Stress Test was performed to monitor OCR. During this test, cells were sequentially treated at specific time points (as indicated in Figure 5c,h) with 1 µm oligomycin (Agilent Technologies), 1.5 µm carbonyl cyanide‐4‐ (trifluoromethoxy) phenylhydrazone (FCCP; Agilent Technologies), and a combination of 0.5 µm rotenone and antimycin A (Rot/AA; Agilent Technologies).

### Flow Cytometry

Bone marrow cells (4 × 10⁶) were treated with RBC lysis and stained with Fixable Viability Stain 780 (1:1000; BD Biosciences) for 15 min at room temperature. Cells were washed with PBS containing 2% BSA (BSA; Gibco) and blocked with anti‐CD16/32 (clone 2.4G2, 1:100; BD Biosciences) for 20 min at 4 °C. The PerCP‐Cy5.5 Mouse Lineage (Lin) Antibody Cocktail (BD Biosciences) was used to label cells from major hematopoietic lineages, including anti‐CD3e (clone 145‐2C11, T cells), anti‐CD11b (clone M1/70, myeloid cells), anti‐CD45R/B220 (clone RA3‐6B2, B cells), anti‐Ly‐76 (clone TER‐119, erythroid cells), and anti‐Ly‐6G and Ly‐6C (clone RB6‐8C5, neutrophils and monocytes).

For further analysis of LKS cells, HSCs, and MPPs, the following antibodies were used: Lin Cocktail‐PerCP‐Cy5.5, anti‐c‐Kit‐BV421 (clone 2B8, BD Biosciences), anti‐Sca‐1‐AF488 (clone D7, Biolegend), anti‐CD150‐AF647 (clone TC15‐12F12.2, Biolegend), anti‐CD48‐BV510 (clone HM48‐1, BD Biosciences), anti‐Flt3‐PE (clone A2F10.1, BD Biosciences), and anti‐CD34‐PE‐Cy7 (clone HM34, Biolegend). Cells were incubated with these antibodies at a 1:100 dilution at 4 °C for 30 min, washed with PBS/2% BSA, and analyzed for the respective markers.

For peripheral blood neutrophils and monocytes, cells were stained with anti‐CD11b‐PE (clone M1/70, Biolegend), anti‐Ly‐6C‐APC (clone AL‐21, BD Biosciences), and anti‐Ly‐6G‐BV421 (clone 1A8, BD Biosciences) at 1:100 dilution for 30 min at 4 °C. After staining, cells were washed and resuspended in 1% paraformaldehyde for fixation.

Data acquisition was performed on a Beckman Coulter DxFLEX flow cytometer using the CytoExpert for DxFLEX software. Data analysis was carried out using FlowJo software (version 10.8.1) to identify and quantify specific cell populations based on predefined gating strategies.

### Biodistribution

Particles were labeled with 1,1'‐Dioctadecyl‐3,3,3',3'‐Tetramethylindodicarbocyanine Perchlorate (DiD; Beyotime Biotechnology) to track their biodistribution. BCG‐DiD (5 × 10^6^ CFU g^−1^ body weight), B‐OMVs‐DiD (5 µg g^−1^ body weight), and equal volumes of PBS and PBS‐DiD (negative and positive controls, respectively) were administered via intraperitoneal (i.p.) or intravenous (i.v.) injection. In vivo imaging was performed at 0, 8, and 24 h post‐injection using a Berthold Technologies NightOWL LB983 imaging system, with analysis conducted using IndiGo software (version 2.0.5.0).

To analyze the biodistribution of B‐OMVs in bone marrow, femurs were collected at 8 or 24 h post‐intraperitoneal injection. Ex vivo imaging of intact femurs was performed using the Berthold Technologies NightOWL LB983 imaging system. Additionally, to visualize B‐OMVs within bone marrow cells, femurs were processed for histological sectioning or used to prepare single‐cell suspensions. For histological analysis, femurs were sectioned and observed under a fluorescence microscope (Olympus). For bone marrow suspension analysis, marrow was flushed from femurs with 1 mL PBS, and the cells were analyzed using a fluorescence microscope (Olympus).

### Safety and Toxicity Evaluation of B‐OMVs

E‐OMVs (5, 7.5, or 10 µg/g body weight) or B‐OMVs (5, 10, 25, or 50 µg/g body weight) were intraperitoneally injected into healthy C57BL/6 mice, with PBS or BCG (5 × 10^4^ CFU g^−1^ body weight) as controls. Mortality was monitored for 7 days. Due to high mortality at 7.5 and 10 µg g^−1^ of E‐OMVs, subsequent experiments used E‐OMVs (10 µg g^−1^) or B‐OMVs (5, 10, 25, or 50 µg/g), with PBS and BCG as controls. Body weights were recorded over 7 days. Blood samples collected 24 h post‐injection were stored at ‐80 °C for analysis. Mice were euthanized to harvest liver and lung tissues for H&E staining.

To assess B‐OMVs infectivity, healthy mice received intraperitoneal injections of B‐OMVs (50 µg g^−1^), E‐OMVs (5 µg g^−1^), BCG (5 × 10^4^ CFU g^−1^), or PBS. At week 3, spleens were harvested and photographed, and lungs were subjected to acid‐fast bacilli staining.

### TLR2‐Dependent In Vitro Trained Immunity Induction by B‐OMVs

B‐OMVs (5 µg mL^−1^) or B‐OMVs (5 µg mL^−1^) + C29 (100 µm, InvivoGen) were used to induce trained immunity. C29 was added 1 h prior to B‐OMVs treatment. PBS or C29 (100 µm) alone were used as controls. All other experimental procedures followed the protocol for in vitro trained immunity induction.

### Quantification Assay

The concentrations of TNF‐α and IL‐6 in mouse serum or cell supernatants were measured using commercial ELISA kits (DAKEWE) following the manufacturer's instructions. The levels of AST, creatinine (CR), and lactate dehydrogenase (LDH) were determined using an automatic biochemical analyzer (Hitachi High‐Tech).

Lactate concentration in the supernatant was quantified using the Lactic Acid Content Assay Kit (Solarbio) according to the manufacturer's protocol. Glucose concentration was measured using the Glucose Assay Kit with O‐toluidine (Beyotime Biotechnology), following the provided instructions.

### Western Blot Analysis

Protein expression was assessed using Western blot analysis. Briefly, samples were lysed in RIPA buffer (Beyotime Biotechnology) to extract total protein. The lysates were centrifuged, and the supernatants were collected for further analysis. Protein samples were separated by 4–12% SDS‐PAGE and transferred onto a PVDF membrane (Millipore). The membrane was blocked with 5% nonfat milk at room temperature for 2 h, followed by overnight incubation at 4 °C with the respective primary antibodies. After washing, the membrane was incubated with secondary antibodies for 1 h at room temperature. Protein bands were visualized using enhanced chemiluminescence (ECL) reagent (Thermo Fisher).

### Endotoxin Assay

The B‐OMVs and E‐OMVs solutions with equivalent concentrations (500 µg mL^−1^) were subjected to three cycles of freeze‐thaw treatment, followed by ultrasonic disruption. The lysates were subsequently centrifuged at 20,000 × g to collect the supernatant. The endotoxin concentration in the supernatant was quantitatively analyzed using a Chromogenic Limulus Amebocyte Lysate (LAL) Endotoxin Assay Kit (Beyotime Biotechnology, Cat#: C0276S) according to the manufacturer's protocol.

### ATAC‐Seq

The ATAC‐seq library was prepared using the Hyperactive ATAC‐Seq Library Prep Kit for Illumina (Vazyme, TD711) according to the manufacturer's instructions. For each sample, 5 × 10^5 cells were collected, washed with 500 µl of PBS, and centrifuged at 500 × g for 5 min at room temperature. The cell pellets were resuspended in 50 µl of cold lysis buffer and incubated on ice for 10 min to release the nuclei. After centrifugation at 500 × g for 5 min at 4 °C, the nuclei were collected and mixed with 50 µl of Tn5 transposome/transposition reaction mix. The reaction was carried out at 37 °C for 30 min to complete the transposition. The fragmented and transposed DNA was purified using VAHTS DNA Clean Beads, followed by two washes with 200 µl of fresh 80% ethanol. The DNA was then eluted in 26 µl of nuclease‐free ddH_2_O.

Library amplification was performed as follows: 72 °C for 3 min, 95 °C for 3 min, 12–15 cycles of 98 °C for 10 s, 60 °C for 5 s, 72 °C for 1 min, and a final hold at 12 °C. The amplified ATAC‐seq library was further purified using VAHTS DNA Clean Beads, washed twice with 200 µl of fresh 80% ethanol, and eluted in 22 µl of nuclease‐free ddH2O. Sequencing was performed on the Illumina NovaSeq 6000 platform.

Sequencing data underwent quality control using FastQC (v0.11.9) to assess read quality. The 150 bp paired‐end reads were aligned to the mouse reference genome (mm10) using standard alignment parameters. Differentially accessible chromatin regions were identified using the DiffBind R package, and differential enrichment peaks were visualized with Integrative Genomics Viewer (IGV).

### Statistical Analysis

All experiments were performed with at least three independent biological replicates. Data are presented as means ± SD, as indicated in the figure legends. The significance of differences between groups was evaluated using one‐way or two‐way analysis of variance (ANOVA), as specified in the figure legends. Statistical significance was considered when *P* < 0.05. Survival analysis was conducted using the log‐rank (Mantel‐Cox) test.

## Conflict of Interest

The authors declare no conflict of interest.

## Author Contributions

Y.G., W.H. and L.X. contributed equally to this work. H.Z. and J.W. conceived and designed the experiments. Y.G., W.H., and L.X. performed most of the experiments and analyzed the data. Z.D., Y.Y., P.P., Z.B., J.H., and Q.S. assisted with some experiments. H.Z., W.H., D.T. and J.H.W. wrote the manuscript. R.K. assisted with data interpretation and edited the manuscript. Z.Z. and J.W. supervised the biological research and study design.

## Supporting information



Supporting Information

## Data Availability

The data that support the findings of this study are available from the corresponding author upon reasonable request.
