# Supplementary material for: BCG‐Derived Outer Membrane Vesicles Induce TLR2‐Dependent Trained Immunity to Protect Against Polymicrobial Sepsis
Source: Adv Sci (Weinh). 2025 Jun 24;12(37):e04101. doi: 10.1002/advs.202504101 (PMC12499445; doi:10.1002/advs.202504101)
Supplement: Supplementary file 1 — Supporting Information [file ADVS-12-e04101-s001.docx]

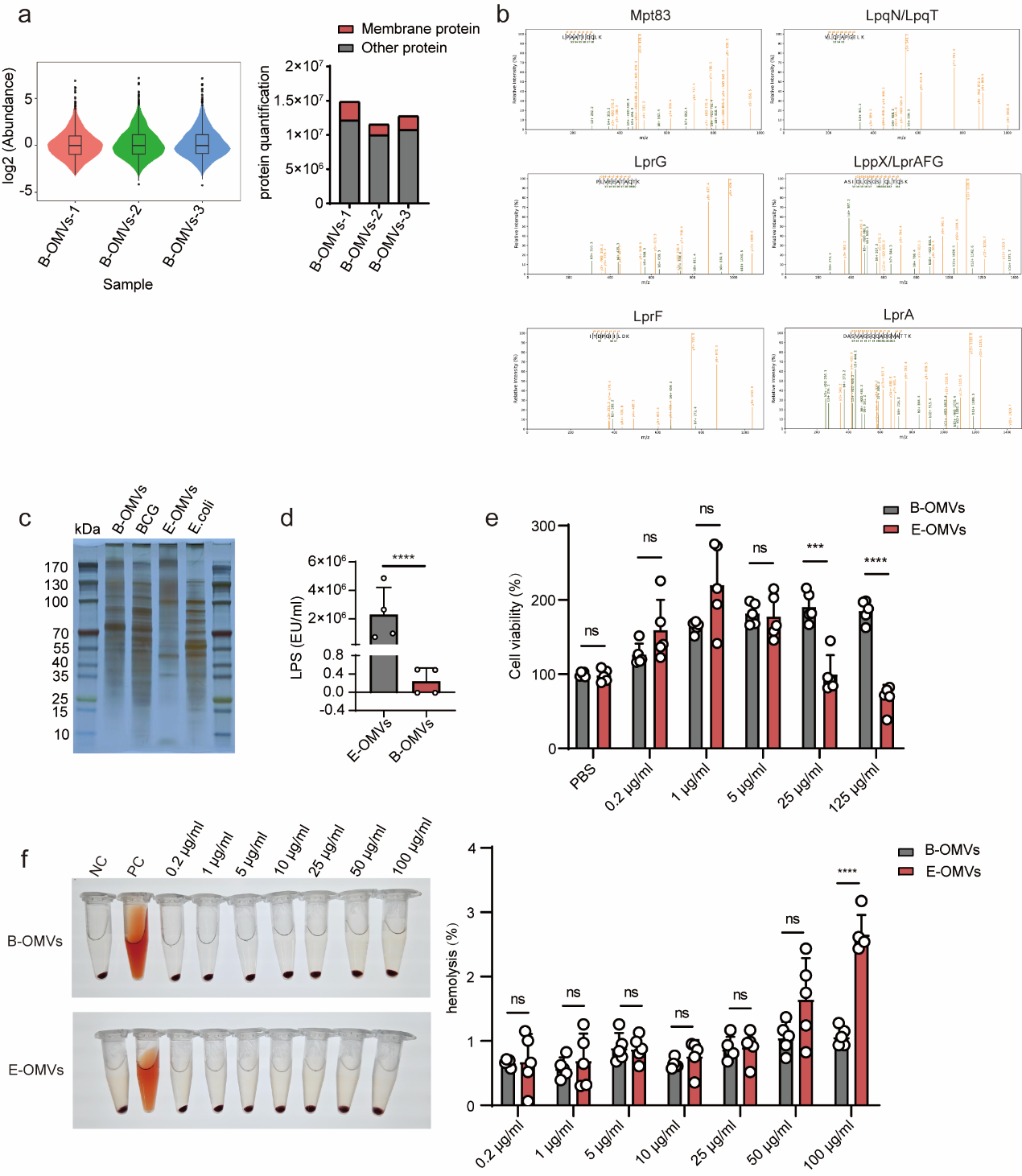


**Figure S1. Characteristic and cytotoxicity of B-OMVs.**

**a)** Proteomic sequencing analysis of B-OMVs.

**b)** The mass spectrometric analysis results of the major protein components in B-OMVs.

**c)** Silver-stained SDS-PAGE analysis of B-OMVs, BCG, E-OMVs and *E.coli*.

**d)** Quantification of endotoxin levels in B-OMVs and E-OMVs at equivalent concentrations (500 μg/mL) using the Chromogenic Limulus Amebocyte Lysate (LAL) Endotoxin Assay Kit (n = 4).

**e)** Cell viability after treatment with the indicated doses of B-OMVs and E-OMVs was detected using the CCK-8 assay (n = 5).

**f)** Hemolytic activity evaluation of the indicated doses of B-OMVs and E-OMVs (n = 5).

Data are presented as means ± SD. Statistical significance was analysed using two-tailed unpaired Student’s t-test (**d-f**). ns, not significant; **p*<0.05, ***p*<0.01, ****p*<0.001, and *****p*<0.001.


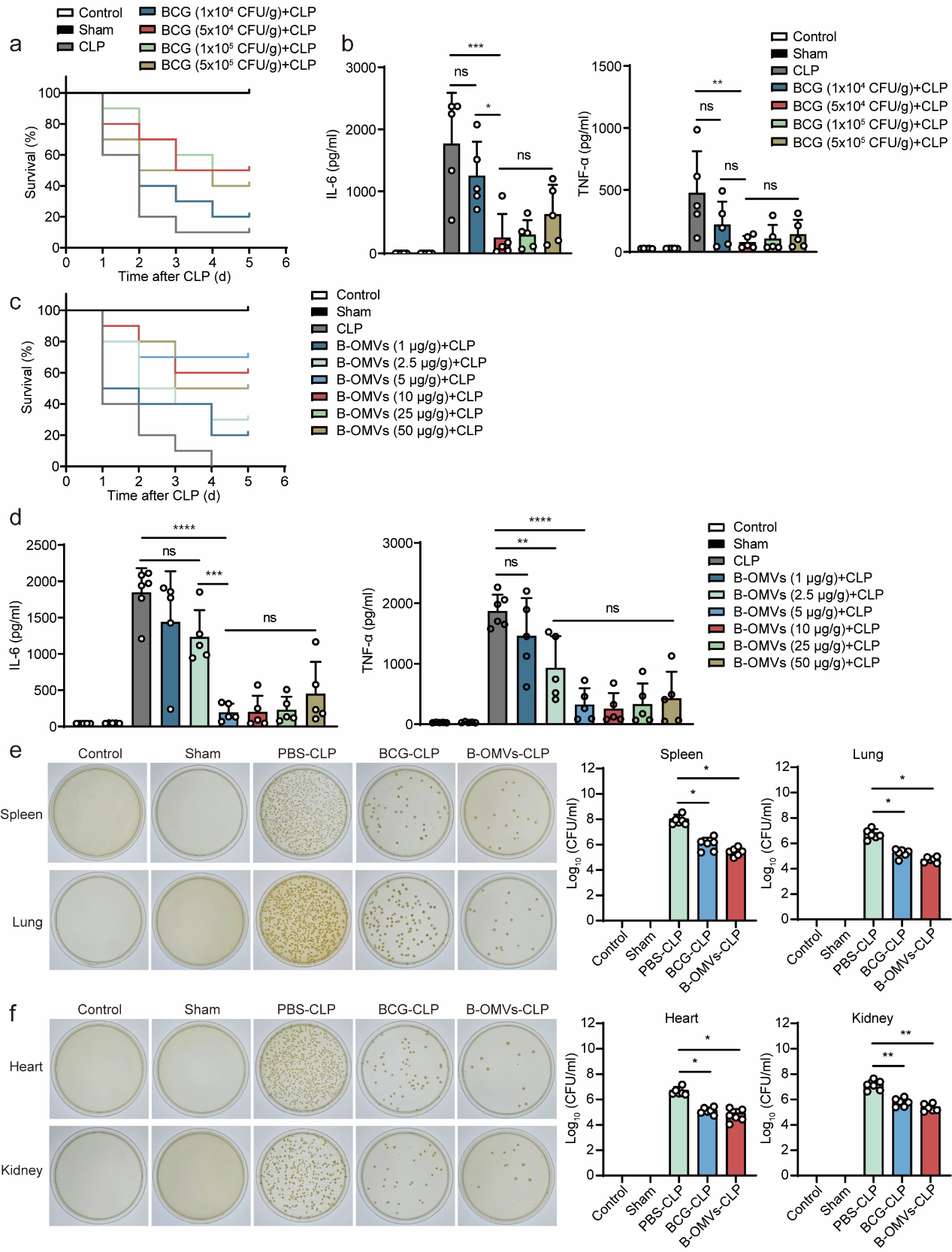


**Figure S2.** **B-OMVs reduce bacterial loads in visceral organs induced by microbial sepsis.**

**a-b)** 6-8-week-old mice were treated intraperitoneally with PBS (Control) or varying doses of BCG (1×10^4^ CFU/g, 5×10^4^ CFU/g, 1×10^5^ CFU/g, 5×10^5^ CFU/g). After a 3-day resting period, the mice underwent CLP to induce sepsis.

**a)** Survival rate of mice in the indicated groups after CLP (n = 10 mice per group).

**b)** Serum levels of IL-6 and TNF-𝛼 in mice from the indicated groups 24 hours after CLP (n = 5 mice per group).

**c-d)** 6-8-week-old mice were treated intraperitoneally with PBS (Control) or varying doses of B-OMVs (1 μg/g, 2.5 μg/g, 5 μg/g, 10 μg/g, 25 μg/g, 50 μg/g). After a 3-day resting period, the mice underwent CLP to induce sepsis.

**c)** Survival rate of mice in the indicated groups after CLP (n = 10 mice per group).

**d)** Serum levels of IL-6 and TNF-𝛼 in mice from the indicated groups 24 hours after CLP (n = 5-6 mice per group).

**e–f**) To evaluate bacterial clearance, spleen, lung, heart, and kidney tissues were collected 24 hours after CLP from mice treated with PBS, BCG (5×10⁴ CFU/g), or B-OMVs (5 μg/g).

**e)** Bacterial loads of spleen and lung from the indicated groups 24 hours after CLP. Representative plates were shown (left panel). Bacterial colonies were quantified (right panel) (n = 6 mice per group).

**f)** Bacterial loads of heart and kidney from the indicated groups 24 hours after CLP. Representative plates were shown (left panel). Bacterial colonies were quantified (right panel) (n = 6 mice per group).

Data are presented as means ± SD. Statistical significance was analysed using one-way ANOVA with Dunnett’s multiple comparisons test (**b, d, e** and **f**). ns, not significant; **p*<0.05, ***p*<0.01, ****p*<0.001, and *****p*<0.001.


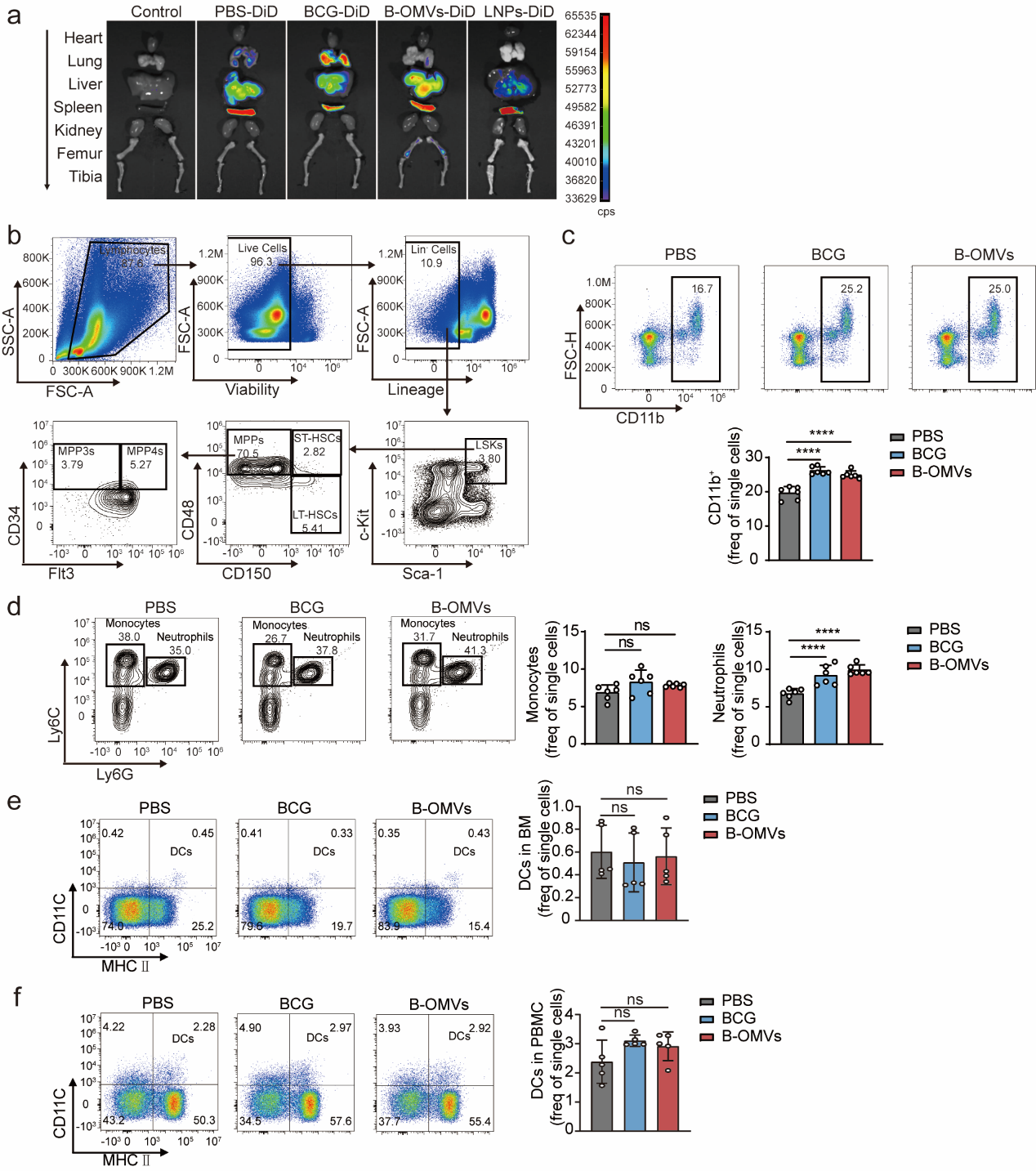


**Figure S3. B-OMVs increase the proportion of myeloid leukopoiesis.**

**a)** Biodistribution of DiD-labeled BCG, B-OMVs and lipid nanoparticles (LNPs) in major organs following intraperitoneal injection. PBS and DiD-labeled PBS (PBS-DiD) served as negative and positive controls.

**b)** Gating strategies for flow cytometry analysis.

**c-f)** 6-8-week-old mice were treated with PBS (control), BCG (5×10^4^ CFU/g), or B-OMVs (5 μg/g). After a 3-day resting period, bone marrow cells and peripheral blood were collected for flow cytometry analysis.

**c)** Flow cytometry analysis of myeloid leukopoiesis in peripheral blood from the indicated groups. The frequencies were quantified (right panel) (n = 6 mice per group).

**d)** Flow cytometry analysis of neutrophils and monocytes in peripheral blood from the indicated groups. The frequencies were quantified (right panel) (n = 6 mice per group).

**e)** Flow cytometry analysis of DCs in bone marrow from the indicated groups. The frequencies were quantified (n = 5 mice per group).

**f)** Flow cytometry analysis of DCs in peripheral blood from the indicated groups. The frequencies were quantified (n = 5 mice per group).

Data are presented as means ± SD. Statistical significance was analysed using one-way ANOVA with Dunnett’s multiple comparisons test (**c**-**f**). ns, not significant; **p*<0.05, ***p*<0.01, ****p*<0.001, and *****p*<0.001.


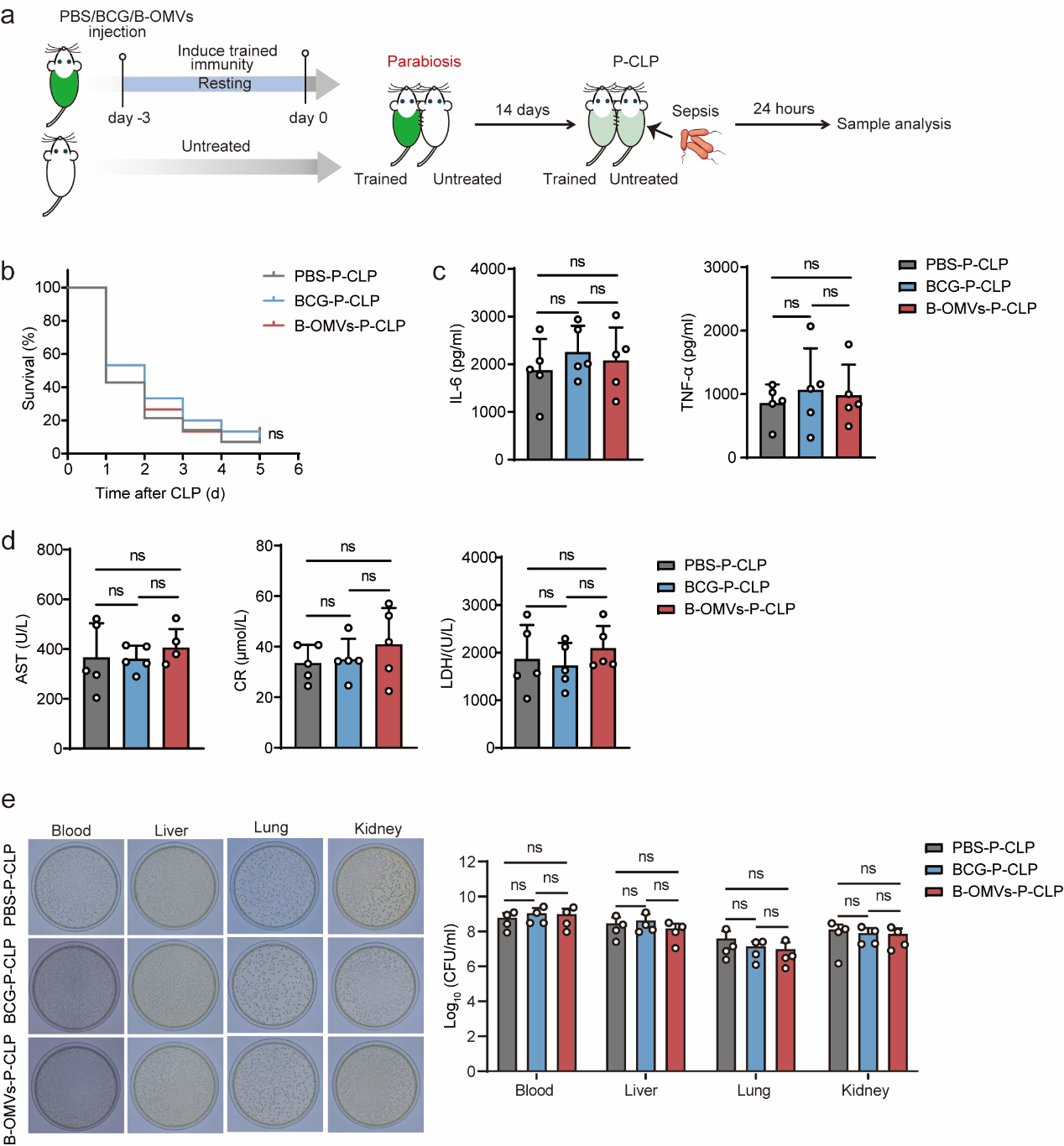


**Figure S4. B-OMVs fail to confer Sepsis protection via parabiosis.**

**a)** Schematic illustration of the parabiosis experiment. Mice were pretreated with PBS, BCG (5×10⁴ CFU/g), or B-OMVs (5 μg/g), then surgically joined with untreated parabionts to establish shared circulation. After two weeks, the untreated partners were subjected to CLP to induce sepsis (P-CLP).

**b)** Survival rate of mice in the indicated groups after CLP (sham: n = 8 mice per group, CLP: n = 14 - 15 mice per group).

**c)** Serum levels of IL-6 and TNF-𝛼 in mice from the indicated groups 24 hours after CLP (n = 5 mice per group).

**d)** Blood biochemistry analysis of AST, CR and LDH levels in serum of mice from the indicated groups 24 hours after CLP (n = 5 mice per group).

**e)** Bacterial loads of blood, liver, lung and kidney from the indicated groups 24 hours after CLP (n = 4 mice per group).

Data are presented as means ± SD. Statistical significance was analysed using one-way ANOVA with Dunnett’s multiple comparisons test (**c-e**) and Log-rank (Mantel-Cox) test (**b**). ns, not significant; **p*<0.05, ***p*<0.01, ****p*<0.001, and *****p*<0.001.


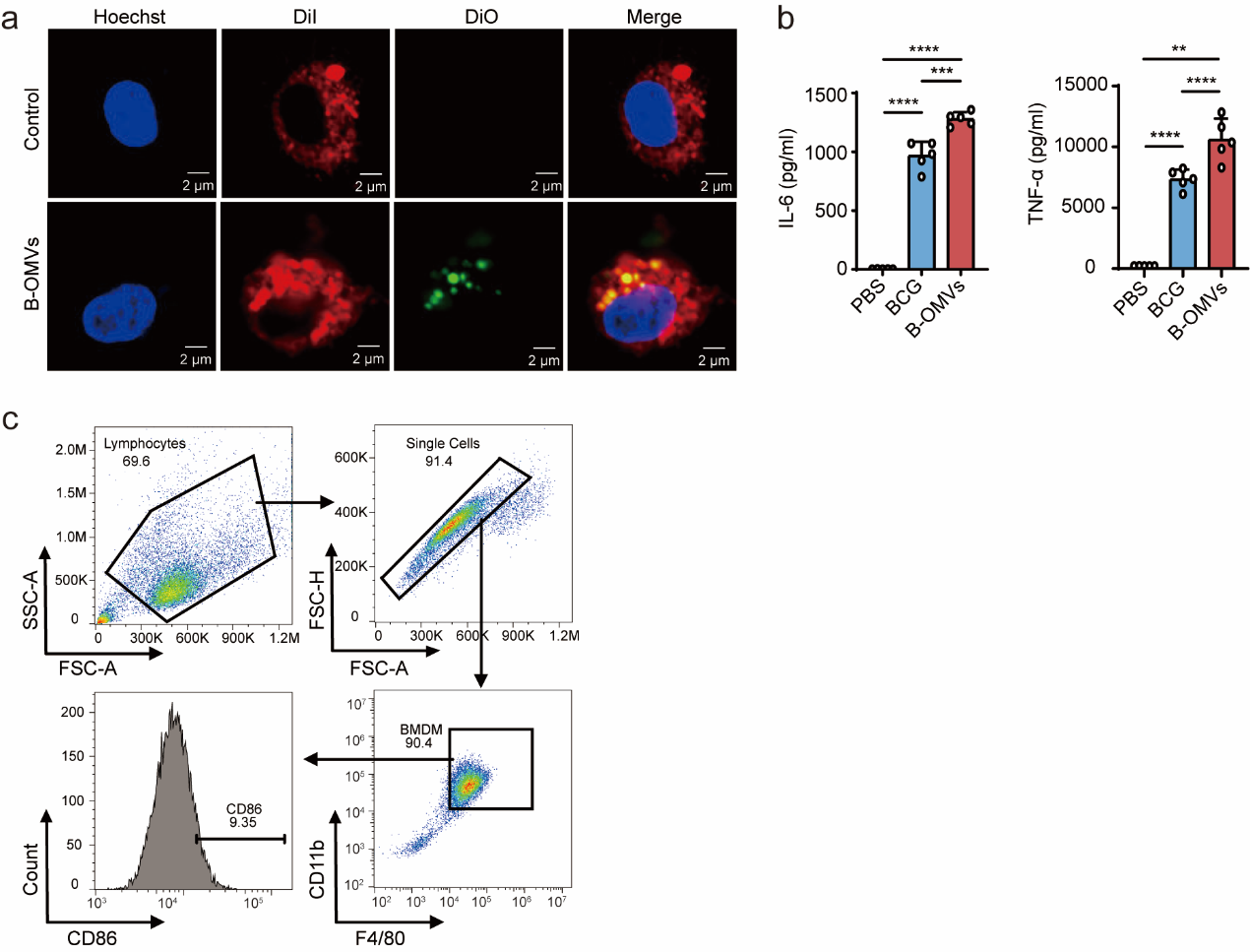


**Figure S5. B-OMVs interact with BMDMs and promote inflammatory responses.**

**a)** Immunofluorescence analysis of BMDMs and B-OMVs. 5 μg/ml DiO-labeled B-OMVs (green) were co-cultured with Dil-labeled BMDMs (red) for 2 hours and co-localization were examined by laser confocal microscopy. Scale bar: 2 μm.

**b)** Levels of IL-6 and TNF-𝛼 in the supernatant. BMDMs were treated with PBS (control), BCG (1×10^4^ CFU/ml) and B-OMVs (5 μg/ml) for 24 hours and the supernatant was collected to assess the levels of IL-6 and TNF-𝛼 (n = 5).

**c)** Gating strategy for flow cytometric analysis of CD86⁺ macrophages.

Data are presented as means ± SD. Statistical significance was analysed using one-way ANOVA with Dunnett’s multiple comparisons test (**b**). ns, not significant; **p*<0.05, ***p*<0.01, ****p*<0.001, and *****p*<0.001.


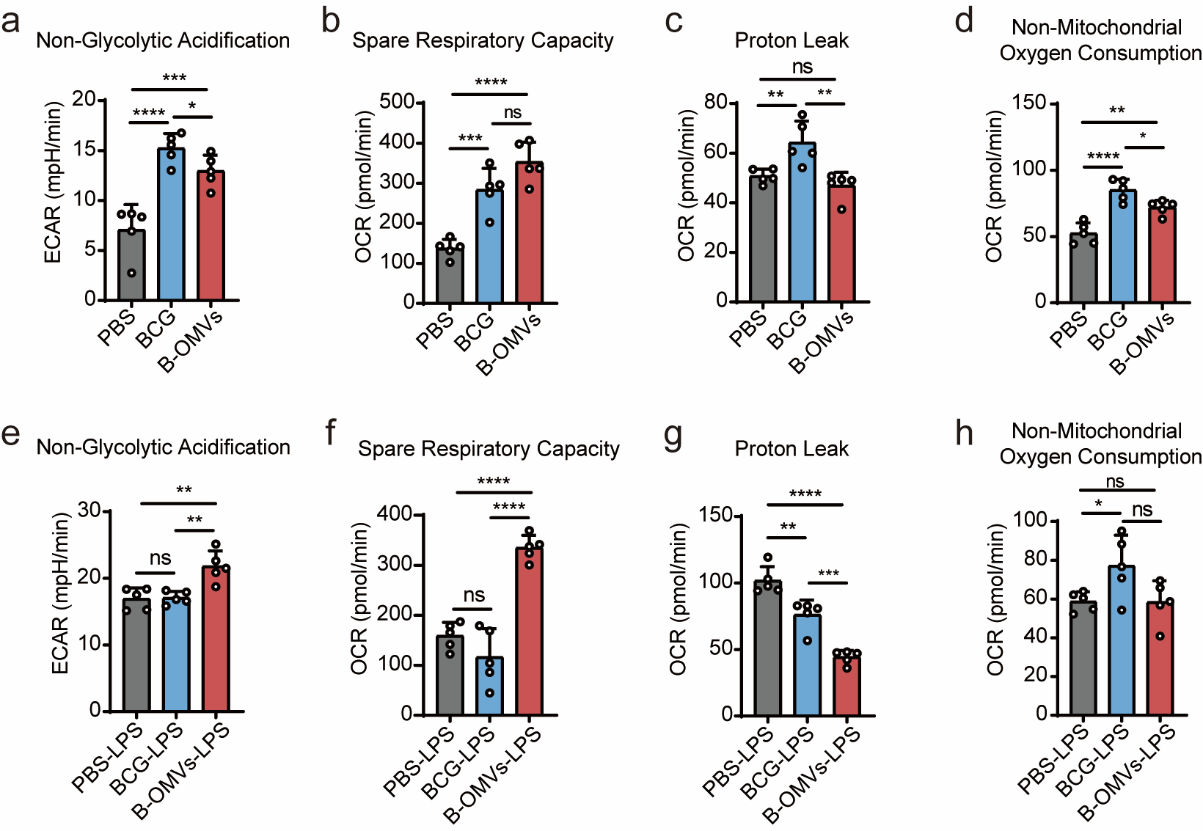


**Figure S6. B-OMVs activate aerobic glycolysis of BMDMs.**

**a-d)** BMDMs were treated with PBS (control), BCG (1×10^4^ CFU/ml) and B-OMVs (5 μg/ml) for 24 hours, followed by a 3-day resting period. Seahorse XF technology was used to evaluate metabolic changes in aerobic glycolysis (a) and OXPHOS (b-d) (n = 5).

**a)** The levels of Non-Glycolytic Acidification of BMDMs from the indicated groups (n = 5).

**b-d)** The levels of Non-Glycolytic Acidification, Spare Respiratory Capacity, Proton Leak and Non-Mitochondrial Oxygen Consumption of BMDMs from the indicated groups (n = 5).

**e-h)** BMDMs were treated with PBS (control), BCG (1×10^4^ CFU/ml) and B-OMVs (5 μg/ml) for 24 hours. After a 3-day resting period, the cells were stimulated by LPS (100 ng/ml) for 12 hours. Seahorse XF technology was used to evaluate metabolic changes in aerobic glycolysis (e) and OXPHOS (f-h).

**a)** The levels of Non-Glycolytic Acidification of BMDMs from the indicated groups after LPS stimulation (n = 5).

**b-d)** The levels of Non-Glycolytic Acidification, Spare Respiratory Capacity, Proton Leak and Non-Mitochondrial Oxygen Consumption of BMDMs from the indicated groups after LPS stimulation (n = 5).

ECAR, extracellular acidification rate; OCR, oxygen consumption rate. Data are presented as means ± SD. Statistical significance was analysed using one-way ANOVA with Dunnett’s multiple comparisons test (**b**, **d**, **g**, **i** and **k**). ns, not significant; **p*<0.05, ***p*<0.01, ****p*<0.001, and *****p*<0.001.


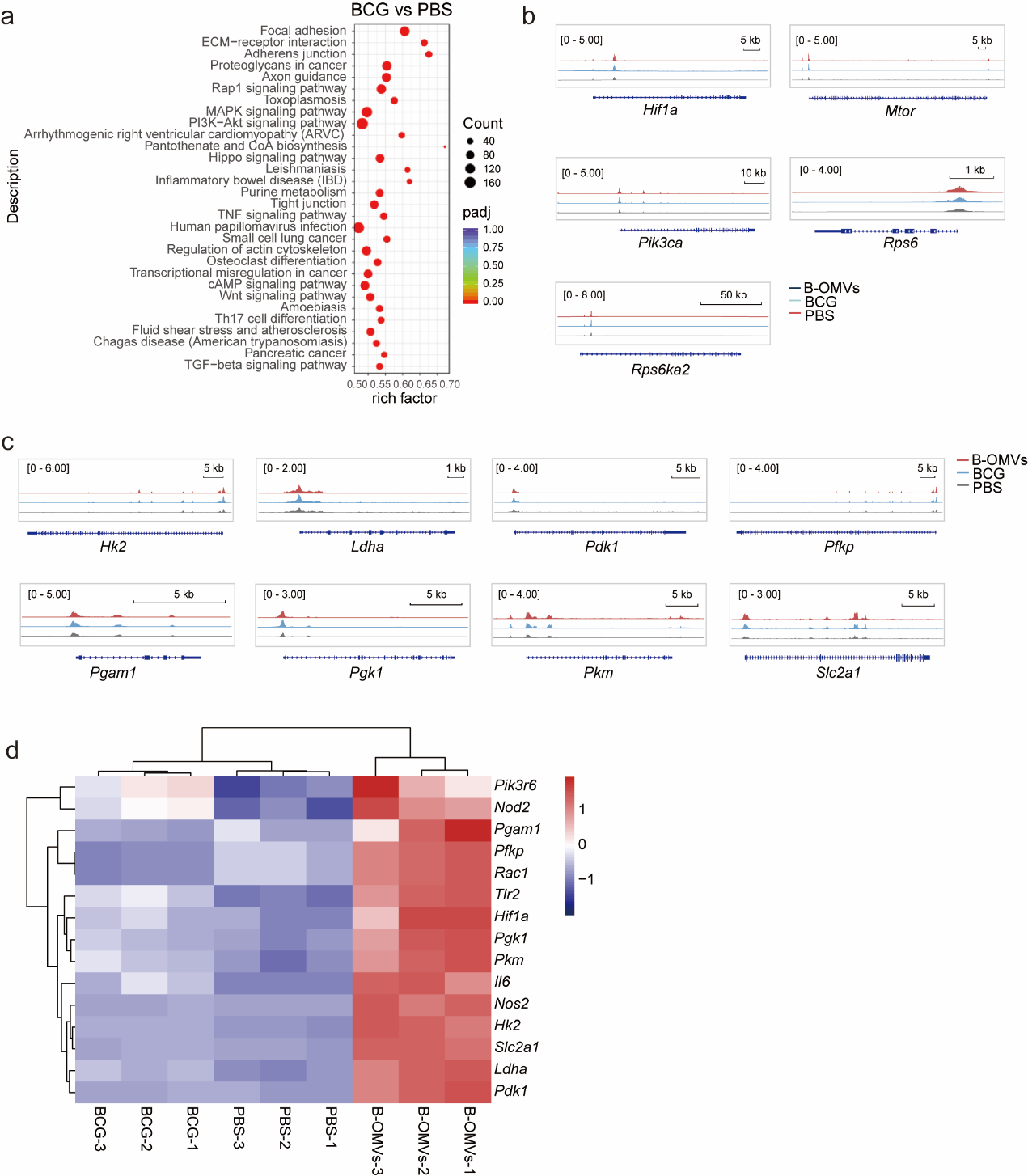


**Figure S7. The epigenetic landscape of BMDMs after B-OMVs training.**

**a)** Heatmap comparison between PBS-treated group and BCG-treated groups. KEGG enrichment analysis of genes with differential ATAC peaks.

**b)** ATAC signals of genes involved in pathways of the Akt-mTOR-HIF axis (*Hif1a*, *Mtor*, *Pik3ca*, *Rps6*, and *Rps6ka2*).

**c)** ATAC signals of genes involved in glycolysis (*Hk2*, *Ldha*, *Pdk1*, *Pfkp*, *Pgam1*, *Pgk1*, *Pkm*, and *Slc2a1*).

**d)** RNA-seq validation of B-OMVs-induced epigenetic reprogramming in glycolysis and immunity.


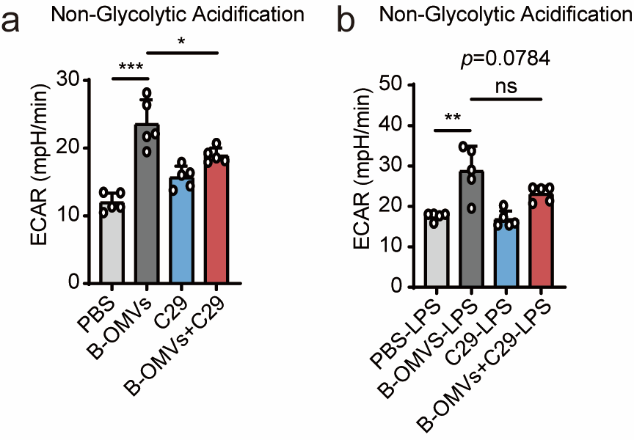


**Figure S8. TLR2 inhibitor C29 decreases B-OMVs-induced aerobic glycolysis.**

**a-b)** BMDMs were treated with PBS (control), B-OMVs (5 μg/ml), C29 (100 μM) as well as B-OMVs (5 μg/ml) and C29 (100 μM) for 24 hours. After a 3-day resting period, the cells were stimulated by PBS (control) or LPS (100 ng/ml) for 12 hours. Seahorse XF technology was used to evaluate glycolytic metabolism.

**a)** The levels of Non-Glycolytic Acidification of BMDMs from the indicated groups (n = 5).

**b)** The levels of Non-Glycolytic Acidification of BMDMs from the indicated groups after LPS stimulation (n = 5).

ECAR, extracellular acidification rate. Data are presented as means ± SD. Statistical significance was analysed using one-way ANOVA with Dunnett’s multiple comparisons test (**a** and **b**). ns, not significant; **p*<0.05, ***p*<0.01, ****p*<0.001, and *****p*<0.001.

**Table S1. Key resource**

| REAGENT or RESOURCE | SOURCE | IDENTIFIER |
| --- | --- | --- |
| Antibodies | | |
| anti-CD16/32 (clone 2.4G2） | BD Biosciences | Cat# 553141 |
| Fixable Viability Stain 780 | BD Biosciences | Cat# 565388 |
| Lin Cocktail-PerCP-Cy5.5 | BD Biosciences | Cat# 561317 |
| anti-c-Kit-BV421 (clone 2B8) | BD Biosciences | Cat# 562609 |
| anti-CD48-BV510 (clone HM48-1) | BD Biosciences | Cat# 563536 |
| anti-Flt3-PE (clone A2F10.1) | BD Biosciences | Cat#553842 |
| anti-Ly-6C-APC (clone AL-21) | BD Biosciences | Cat#560595 |
| anti-Ly-6G-BV421 (clone 1A8) | BD Biosciences | Cat#562737 |
| anti-Sca-1-AF488 (clone D7) | Biolegend | Cat#108116 |
| anti-CD150-AF647 (clone TC15-12F12.2) | Biolegend | Cat#115918 |
| anti-CD34-PE-Cy7 (clone HM34) | Biolegend | Cat#128618 |
| anti-CD11b-PE (clone M1/70) | Biolegend | Cat#101208 |
| anti-F4/80-FITC (clone BM8) | Biolegend | Cat#123108 |
| anti-CD86-PE-Cy7(clone GL1) | eBioscience | Cat#25-0862-82 |
| Rabbit monoclonal anti-mTOR (7C10) | Cell Signaling Technology | Cat#2983 |
| Phospho-mTOR (Ser2448) | Cell Signaling Technology | Cat#2971 |
| Rabbit monoclonal anti-Akt(C67E7) | Cell Signaling Technology | Cat#4691 |
| Phospho-Akt (Ser473) | Cell Signaling Technology | Cat#4060 |
| Rabbit polyclonal anti-HIF-1α | Wuhan Sanying Biotechnology | Cat#20960-1-AP |
| Monoclonal Mouse Anti-β-Actin antibody | Wuhan Sanying Biotechnology | Cat#66009-1-Ig |
| Goat anti-Mouse IgG (H + L) Secondary Antibody, HRP | Jackson ImmunoResearch | Cat#115-035-003 |
| Goat anti-Rabbit IgG (H + L) Secondary Antibody, HRP | Jackson ImmunoResearch | Cat#111-035-003 |
| Rabbit polyclonal anti-TLR2 | ABclonal Biotechnology | Cat#A2545 |
| Goat anti-Rabbit IgG (H+L) Secondary Antibody, ABflo594 | ABclonal Biotechnology | Cat#AS039 |
|  |  |  |
| Chemicals, Peptides, and Recombinant Proteins | | |
| Hoechst 33342 | Beyotime Biotechnology | Cat#C1029 |
| DiO | Beyotime Biotechnology | Cat#C1993S |
| DiI | Beyotime Biotechnology | Cat#C1991S |
| DiD | Beyotime Biotechnology | Cat#C1995S |
| Lipopolysaccharide from Escherichia coli | Sigma-Aldrich | Cat#L2880 |
| XF Cell Mito Stress Test Kit | Agilent Technologies | Cat#103015-100 |
| XF Glycolysis Stress Test Kit | Agilent Technologies | Cat#103020-100 |
| XFe24 FluxPak | Agilent Technologies | Cat#102340-100 |
| XF DMEM Base Medium | Agilent Technologies | Cat#103575-100 |
| XF 1.0 M Glucose Solution | Agilent Technologies | Cat#103577-100 |
| XF 100 mM Pyruvate Solution | Agilent Technologies | Cat#103578-100 |
| XF 200 mM Glutamine Solution | Agilent Technologies | Cat#103579-100 |
| pHrodo Green BioParticles Phagocytosis Kit | Invitrogen | Cat#P35381 |
| Middlebrook 7H10 | Solarbio | Cat#LA7230 |
| Middlebrook 7H9 | Solarbio | Cat#LA7220 |
| OADC | Solarbio | Cat#LA9560 |
| ADC | Solarbio | Cat#LA0272 |
| Tween-80 | Solarbio | Cat#T8360 |
| glycerol | Solarbio | Cat#G8190 |
| Fetal Bovine Serum | Gibco | Cat#A5670801 |
| M-CSF | Stemcell | Cat#78059 |
| Bacterial Strains | | |
| BCG | Chengdu Institute of Biological Products Co. Ltd (Chengdu, China) | N/A |
| *E. coli* | ATCC | Cat#25922 |
| FITC-labeled *E. coli* | Generously gifted by Professor Liao Yuhui, Southern Medical University (Guangdong, China) | N/A |
